# The *Caenorhabditis elegans* cuticle and precuticle: a model for studying dynamic apical extracellular matrices in vivo

**DOI:** 10.1093/genetics/iyae072

**Published:** 2024-07-12

**Authors:** Meera V Sundaram, Nathalie Pujol

**Affiliations:** Department of Genetics, University of Pennsylvania Perelman School of Medicine, Philadelphia, PA 19104, USA; Aix Marseille University, INSERM, CNRS, CIML, Turing Centre for Living Systems, 13009 Marseille, France

**Keywords:** *C. elegans*, cuticle, extracellular matrix, collagen, WormBook, ZP

## Abstract

Apical extracellular matrices (aECMs) coat the exposed surfaces of animal bodies to shape tissues, influence social interactions, and protect against pathogens and other environmental challenges. In the nematode *Caenorhabditis elegans*, collagenous cuticle and zona pellucida protein-rich precuticle aECMs alternately coat external epithelia across the molt cycle and play many important roles in the worm's development, behavior, and physiology. Both these types of aECMs contain many matrix proteins related to those in vertebrates, as well as some that are nematode-specific. Extensive differences observed among tissues and life stages demonstrate that aECMs are a major feature of epithelial cell identity. In addition to forming discrete layers, some cuticle components assemble into complex substructures such as ridges, furrows, and nanoscale pillars. The epidermis and cuticle are mechanically linked, allowing the epidermis to sense cuticle damage and induce protective innate immune and stress responses. The *C. elegans* model, with its optical transparency, facilitates the study of aECM cell biology and structure/function relationships and all the myriad ways by which aECM can influence an organism.

## Introduction to the cuticle and precuticle

While the cuticle has long been recognized as a defining feature of nematodes, the precuticle was discovered more recently. Much progress has been made in understanding both of these aECMs since the last Wormbook review of this topic (Page and Johnstone 2007). It now is clear that the precuticle and cuticle play numerous important roles in *Caenorhabditis elegans* biology and that they share both functional and molecular properties with mammalian aECMs. Here, we provide a comprehensive overview of the contents, structure, and dynamics of these two aECMs and their functions in *C. elegans* development, behavior, and physiology.

### *Caenorhabditis elegans* as a model for studying apical extracellular matrix

All exposed epithelial surfaces on animal bodies are coated by some type of aECM that shapes tissues and serves as the first line of defense against environmental challenges. Apical ECMs are molecularly distinct from stromal ECMs or basement membranes, which are found on the opposite side of polarized epithelia, and they are more diverse in their composition and structure across tissues and organisms. For example, aECMs in humans include the keratin- and lipid-rich stratum corneum of the skin (Feingold and Elias 2014), the mucin-rich linings of the upper respiratory tract and gut (Pelaseyed and Hansson 2020), the lipid-rich surfactant of lung alveoli (Sorensen 2018; Agudelo *et al.* 2020), the sugary glycocalyx of the vascular system (Butler *et al.* 2020), and sensory matrices such as the collagenous tectorial membrane of the inner ear (Sellon *et al.* 2019). Bird feathers and reptile scales are aECMs made of keratin, similar to human hair and nails (Parry 2021). Within the Ecdysozoa (molting invertebrates), arthropods like insects and crustaceans have a chitin-based cuticle that is much more rigid than the collagen-based cuticle of nematodes (Aguinaldo *et al.* 1997; Page *et al.* 2014; Fabritius and Moussian 2017; Zheng *et al.* 2020). Partly as a consequence of this huge diversity, the structure and cell biology of many aECMs remain quite poorly understood.

Although diverse, many aECMs do exhibit shared features such as multi-layered organization, high sugar and lipid content, and presence of typical aECM proteins such as Zona Pellucida (ZP) proteins and mucins (Supplementary Table 1; see below (Zheng *et al.* 2020)). Because of its optical transparency, *C. elegans* allows aECM components to be easily visualized with fluorescent fusions in live animals, avoiding the need for chemical fixation that can destroy fragile matrix structures. *Caenorhabditis elegans* also has a rapid life cycle and many available genetic tools for removing or altering gene function. Therefore, *C. elegans* provides a powerful model system for studying the basic cell biology and structure/function relationships of matrix or matrix-adjacent protein families along with the varied biological roles that aECM can play. A detailed understanding of the *C. elegans* aECM also has practical implications for the control of parasitic nematodes (Blaxter *et al.* 1992; Davies and Curtis 2011; Page *et al.* 2014).

### Dynamics of the cuticle and precuticle

**Fig. 1.**
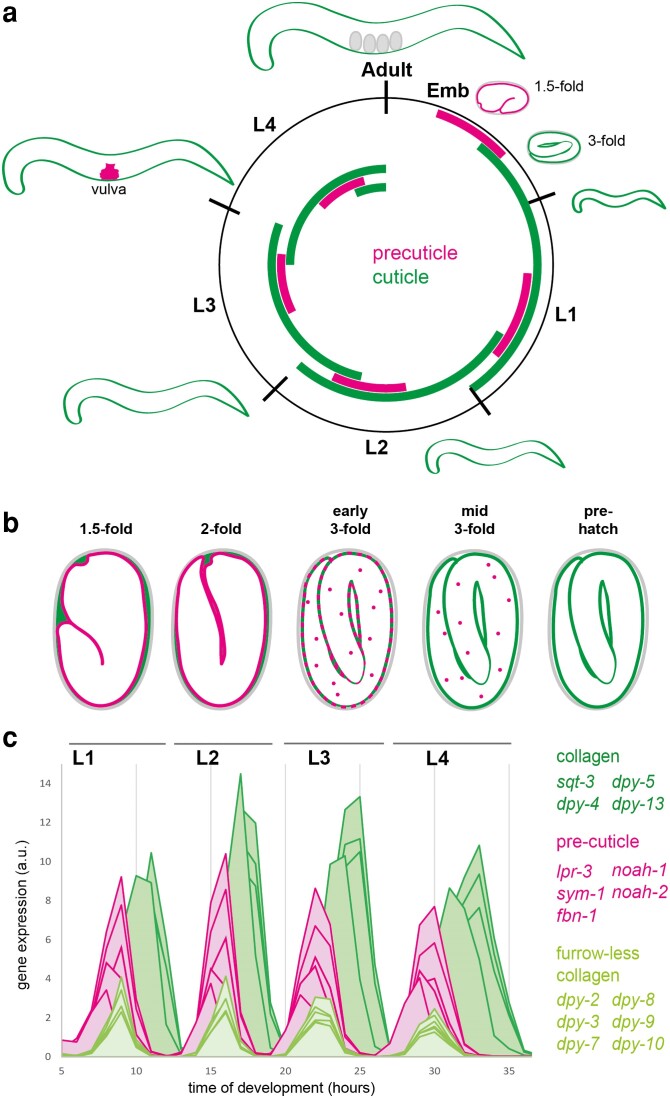
aECM dynamics across the *C. elegans* life cycle. a) The molt cycle. Assembly and removal/shedding of the precuticle (pink) and cuticle (green) are indicated by the inner bars. In larvae, the precuticle and then the new cuticle are assembled underneath the pre-existing cuticle before it is shed. Contrary to cuticle, precuticle is endocytosed and cleared before hatch/molt. Note that precise timing varies among different matrix components and cartoons illustrate only general trends. b) Dynamics of the first precuticle and cuticle in the embryo, based on Birnbaum *et al.* (2023). Cuticle collagens (DPY-17, SQT-3) are initially present in the extraembryonic space, outside of the precuticle (NOAH-1, LPR-3). The precuticle is endocytosed (puncta) after cuticle collagens assemble at the membrane. c) Oscillation of matrix gene expression during the molt cycle. Different groups of precuticle and cuticle genes cycle differently, with precuticle and furrow collagens cycling earlier than annuli collagens; adapted from Meeuse *et al.* (2020).

**Fig. 2.**
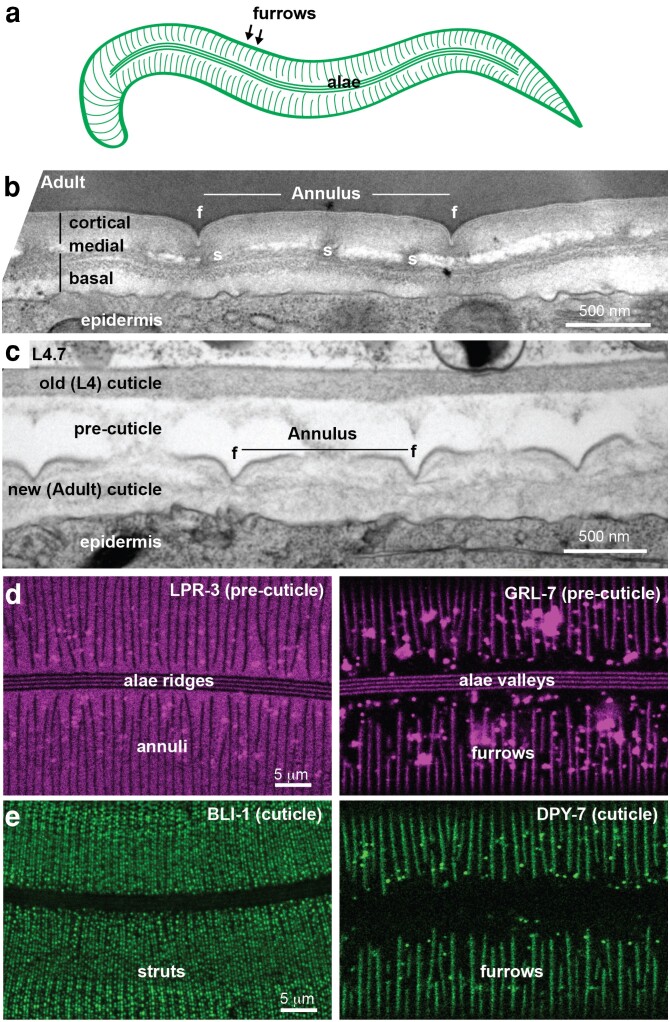
Cuticle substructures. a) Cartoon diagram of adult body cuticle, showing circumferential furrows and longitudinal alae. b) Longitudinal TEM slice of the adult epidermis, showing cuticle annuli/furrows and layers. f, furrow. s, struts. Image credit: Nicolas Brouilly & N.P. c) Longitudinal TEM slice of the epidermis in a L4.7 stage larva. Note the presumed precuticle matrix present between the old and new cuticles. Image credit: Nicolas Brouilly & N.P. d and e) Confocal images of tagged matrix proteins at mid-L4. d) Precuticle examples (SfGFP::LPR-3, left and GRL-7::mCherry, right). e) Cuticle examples (strut collagen BLI-1::mNG, left and furrow collagen DPY-7::SfGFP, right). Image credits: Nicholas Serra, Trevor Barker, and Susanna Birnbaum.

**Fig. 3.**
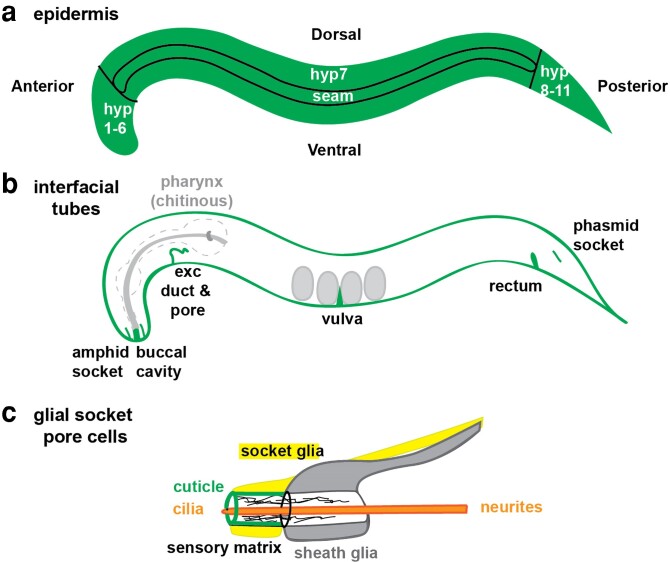
Cuticle-lined epithelia in the adult hermaphrodite. a) The epidermis. Most of the epidermis consists of syncytia generated by cell–cell fusion during embryogenesis (Podbilewicz and White 1994). hyp7 is the epidermal syncytium that encloses most of the body, whereas hyp1–6 are at the anterior and hyp8–11 are at the posterior. The lateral seam epidermis exists as individual cells until the early L4 stage, when these too fuse to form left and right seam syncytia. b) Interfacial tubes. During larval molting, the cuticle linings of these tubes are also shed and replaced. c) Sensilla consist of socket pore (yellow) and sheath (gray) glial cells that form tube-like compartments that surround the distal portions of ciliated sensory neurons, allowing them to access the environment. Socket glia are lined by precuticle and cuticle (green) but also share sensory matrices with the sheath glia. These different matrices help shape the tube channel and connect it to the epidermal cuticle (Perens and Shaham 2005; Heiman and Shaham 2009; Oikonomou *et al.* 2011; Low *et al.* 2019) (*The Precuticle*). In males, some socket glia produce specialized cuticular mating structures (Sulston *et al.* 1980; Jiang and Sternberg 1999) (*The Cuticle*).

The *C. elegans* cuticle lines environment-facing apical surfaces of the epidermis and interfacial tubes (Figs. 1–3). The body cuticle is a flexible exoskeleton decorated with regional substructures, the circumferential furrows and longitudinal alae ridges (Fig. 2), and it plays many different roles in *C. elegans* biology (*The Cuticle*). Because the cuticle is made primarily of collagens, it provides a tractable in vivo system for investigating collagen matrix regulation (*Cuticle collagen maturation*). The first (L1) cuticle is synthesized during late embryogenesis (Sulston *et al.* 1983; Costa *et al.* 1997; Birnbaum *et al.* 2023) (Fig. 1a, b). *Caenorhabditis elegans* molts between its four larval stages (L1–L4) and the adult stage, so it needs to build a new cuticle and shed the old one at four additional times during its life cycle (Figs. 1a and 2b, c). Cuticle structure differs between tissues and developmental stages, and it changes with age, influencing biochemical and mechanical properties and the way that the animal interacts with its environment over its lifetime.

The precuticle is a transient or provisional matrix that precedes the new cuticle and plays important roles in cell and tissue shaping (*The Precuticle*). For example, the precuticle sheath coats epidermal surfaces during embryonic morphogenesis, whereas the cuticle is synthesized only after the embryo has fully elongated (Priess and Hirsh 1986; Mancuso *et al.* 2012; Kelley *et al.* 2015; Gill *et al.* 2016; Vuong-Brender *et al.* 2017; Birnbaum *et al.* 2023) (Figs. 1b and 2d). The precuticle is endocytosed and removed as the cuticle gets built (Birnbaum *et al.* 2023), so that when the L1 larva hatches from the eggshell, the precuticle is no longer present. However, the precuticle reappears before each new cuticle during the molt cycle (Fig. 1a), and it coats the body while postembryonic structures develop and the process of building and exchanging cuticles takes place (Fig. 2d, e). Like the cuticle, precuticle content differs between tissues and developmental stages, but it generally contains various noncollagen components such as ZP domain proteins (Kelley *et al.* 2015; Gill *et al.* 2016; Vuong-Brender *et al.* 2017; Cohen *et al.* 2019). The precuticle and cuticle coexist transiently and can influence each others' structure in ways that we are just beginning to learn (*Future Prospects*).

Because the precuticle and cuticle must be synthesized and cleared in the proper sequence throughout the *C. elegans* life cycle, the genes encoding structural components of these aECMs often are expressed in recognizable oscillatory patterns (Fig. 1c) (Johnstone and Barry 1996; hyun Kim *et al.* 2013; Hendriks *et al.* 2014; George-Raizen *et al.* 2014; Meeuse *et al.* 2020). These patterns are controlled by an oscillatory gene regulatory network (GRN) or “molting clock” involving the Period ortholog LIN-42 (Jeon *et al.* 1999; Monsalve *et al.* 2011), multiple nuclear hormone receptors, and other transcription factors (Stec *et al.* 2021; Tsiairis and Großhans 2021; Hauser *et al.* 2022; Patel *et al.* 2022; Johnson *et al.* 2023; Kinney *et al.* 2023; Meeuse *et al.* 2023). This molting clock must be carefully coordinated with the heterochronic miRNA-based developmental clock, which controls the timing of cell divisions and other stage-specific events (Ruaud and Bessereau 2006; Rougvie and Moss 2013; Patel *et al.* 2022; Kinney *et al.* 2023). A current challenge is to identify the downstream targets of these GRNs in different stages and tissues and to tie them to specific aspects of matrix cell biology discussed here.

It is worth noting that these widespread oscillations in larval gene expression present big challenges for comparative analyses of RNA sequencing data, since even slight differences in staging between two populations can result in large observed differences in gene expression (George-Raizen *et al.* 2014; Tsiairis and Großhans 2021). It is therefore important to assess staging carefully and/or to perform multiple replicates at different timepoints to confirm whether a perturbation impacts gene expression. Computational methods have recently been developed to precisely estimate the real age of a sample from its transcriptome, exploiting existing time-series as reference (Bulteau and Francesconi 2022).

### Which epithelia are lined by precuticle and cuticle?

*Caenorhabditis elegans* epithelia can be divided into three main groups based on the type of aECM that they contain:

1. External epithelia are lined by precuticle and collagen-based cuticle. These include the entire epidermis (hyp1–11 and seam cells) but also interfacial tubes such as the buccal cavity, rectum, excretory duct and pore, and the vulva (Fig. 3a, b). Glial socket pore cells also resemble external epithelia and are lined by precuticle and cuticle (Ward *et al.* 1975; Page and Johnstone 2007; Low *et al.* 2019; Fung *et al.* 2023) (Fig. 3c).
2. The pharynx or foregut tube has a chitin-based cuticle that is very different from the body cuticle (Zhang *et al.* 2005; George-Raizen *et al.* 2014; Kamal *et al.* 2022). The eggshell that surrounds the embryo also is a chitin-based aECM (Zhang *et al.* 2005; Olson *et al.* 2012). These aECMs have been reviewed elsewhere (Stein and Golden 2018; Cohen and Sundaram 2020) and will not be discussed further here.
3. Internal epithelia lack cuticle and instead are lined by other forms of aECM that have been visualized by electron microscopy but are otherwise mostly uncharacterized (Cohen and Sundaram 2020). These epithelia include the intestine, the uterus, and the excretory canal cell. Glial sheath cells also contain sensory matrices that may be shared in part with their attached glial socket cells but appear distinct from precuticle and cuticle (Perens and Shaham 2005; Shaham 2015; Low *et al.* 2019) (Fig. 3c).

### Major protein families found in the cuticle and/or precuticle

**Fig. 4.**
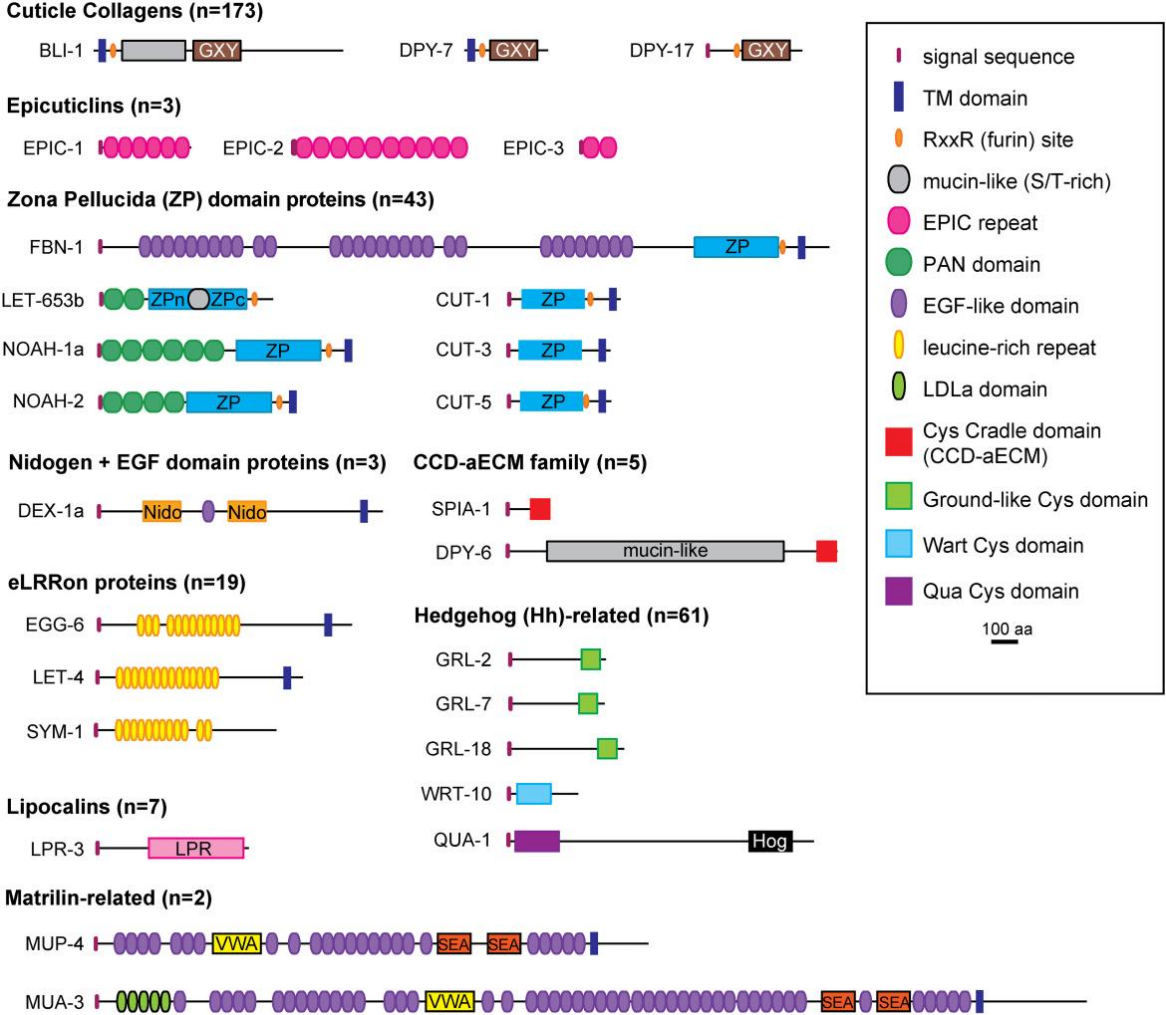
aECM proteins with their domains. Only a few representatives of each matrix protein family are shown. See text and Supplementary Table 1 for further information.

The cuticle and precuticle each contain many protein components that resemble those found in ECMs of humans and other organisms but also some components that appear nematode-specific (Fig. 4, Supplementary Table 1). The following list of matrix components is necessarily incomplete since new components are still being found. For some protein families, different members belong to precuticle vs cuticle. Some specific proteins can even serve as precuticle in one tissue but as cuticle in another. Many matrix factors are present only in certain tissues or stages—see Supplementary Table 1 and Wormbase (Davis *et al.* 2022; Sternberg *et al.* 2024) for details.

#### Cuticle collagens

Collagens are fibril or mesh-forming proteins that are the most abundant structural components of the extracellular matrix in both *C. elegans* and humans. In vertebrates, collagens make up the bulk of stromal matrices and basement membranes, but they are also found in some aECMs such as the tectorial membrane of the inner ear (Sellon *et al.* 2019). Small collagen-related proteins, the collectins, are also found in many aECMs, including lung surfactant (Sorensen 2018; Murugaiah *et al.* 2020). *Caenorhabditis elegans* has at least 173 known or predicted cuticle collagens (Teuscher *et al.* 2019). Most cuticle collagens are relatively small (<350 amino acids) and have interesting similarities to vertebrate transmembrane collagens (*Cuticle Collagen Maturation*). Their large diversity is explained by diverse functions, including as components of specific structures (e.g. furrows, alae—see Fig. 2) or stages (e.g. dauer, adult) (*The Cuticle*). Some collagens could conceivably be part of the transient precuticle, though no such collagens have been identified to date.

#### Cuticlins and epicuticlins

Cuticlins, or CUT proteins, comprise various non-collagen proteins that are found (or suspected to be) within the insoluble fraction of the cuticle of *C. elegans* and/or other nematodes (Fujimoto and Kanaya 1973). These include several ZP proteins (see below) and the low complexity secreted protein CUT-2 (Lassandro *et al.* 1994). The name “CUT-like” or CUTL- has been used for many additional ZP proteins based on homology to cuticlins. The epicuticlins (EPICs) are more recently identified insoluble and intrinsically disordered proteins isolated from an outer cuticle layer of *Ascaris suum*; *Caenorhabditis elegans* has three members of this family, EPIC-1–3 (Bisoffi *et al.* 1996; Betschart *et al.* 2022; Pooranachithra *et al.* 2024). Interestingly, both the ZP and non-ZP cuticlins and epicuticlins have some similarities to matrix factors from arthropods.

#### ZP domain proteins

ZP proteins are fibrillar constituents of aECM in many animal species, including *Drosophila* and vertebrates (Plaza *et al.* 2010). Their name comes from specific proteins (ZP1,2,3) within the zona pellucida matrix layer that coats the mammalian oocyte (Wassarman and Litscher 2021), but ZP proteins also are found in the tectorial membrane of the inner ear (Kim *et al.* 2019) and in luminal matrices of the kidney and vascular system (Negri and Spivacow 2023; Rossi and Bernabeu 2023). *Caenorhabditis elegans* has a large expansion of this family with 43 different ZP-encoding genes (Cohen *et al.* 2019; Weadick 2020). These include components of both the precuticle (NOAH-1, NOAH-2, FBN-1, LET-653) and cuticle (CUT-1, CUT-6, RAM-5), as well as components of other types of aECMs such as sensory matrices (DYF-7). Some of these ZP proteins also contain other matrix domains, such as Plasminogen/Apple (PAN) domains, EGF-like repeats, von Willebrand factor domains, or mucin domains.

ZP domains usually have two halves, ZP-n and ZP-c, which each adopt an immunoglobulin-like fold and can have separable functions (Bokhove and Jovine 2018). The ZP-c portion of LET-653 is sufficient to confer both its localization and its tube-shaping function (Cohen, Bermudez *et al.* 2020). *Caenorhabditis elegans* also has five uncharacterized ZP proteins that contain only a ZP-c portion (Cohen *et al.* 2019; Weadick 2020).

#### Nidogen domain proteins

DEX-1 is an apical transmembrane protein that contains an EGF-like domain and two nidogen domains similar to those found in the conserved basement membrane protein nidogen and the sensory matrix factor tectorin alpha (Heiman and Shaham 2009). It appears to function with a variety of ZP proteins and may be an example of a protein that is found in both precuticle and cuticle: it incorporates into precuticle of specific interfacial tubes and into alae cuticle of the L1 and dauer stages (Heiman and Shaham 2009; Cohen *et al.* 2019; Flatt *et al.* 2019). A paralog (B0393.5) also has aECM-related roles (J. Cohen and MVS, unpublished).

#### Matrilin-related proteins

Vertebrate matrilins are von Willebrand factor type A (VWA) and EGF domain-containing matrix proteins that can bind collagens and proteoglycans and impact ECM organization (Paulsson and Wagener 2018; Li *et al.* 2020). Two *C. elegans* matrilin-like proteins, MUP-4 and MUA-3, are large transmembrane proteins that have been proposed to link the cuticle to the plasma membrane at hemidesmosomes (HDs) (Bercher *et al.* 2001; Hahn and Labouesse 2001; Hong *et al.* 2001).

#### Extracellular leucine-rich repeat only proteins

Extracellular leucine-rich repeat only (eLRRon) proteins are either transmembrane or secreted proteins with extracellular leucine-rich repeats but no other recognizable domains (Dolan *et al.* 2007). This family resembles the collagen-binding small leucine-rich proteoglycans (SLRPs) found in matrices throughout the human body (Kalamajski and Oldberg 2010; Maiti *et al.* 2023). *Caenorhabditis elegans* has 19 members of this family, at least three of which (EGG-6, LET-4, SYM-1) are precuticle components or regulators (Davies *et al.* 1999; Mancuso *et al.* 2012; Vuong-Brender *et al.* 2017). Another member of the family, PAN-1, affects molting (Frand *et al.* 2005; Gissendanner and Kelley 2013), potentially through its role in regulating the membrane-bound transcription factor MYRF-1 (Meng *et al.* 2017; Xia *et al.* 2021; Xu *et al.* 2024). Other eLRRon proteins mediate cell-cell adhesion or cytoskeletal remodeling (Liu and Shen 2011; Zou *et al.* 2018).

#### Lipocalins

Lipocalins are secreted cup-shaped proteins that transport lipids, steroid hormones, or other lipophilic cargo throughout the body and sometimes interact with specific cell surface receptors for cargo delivery or signaling (Steinhoff *et al.* 2021; Schröder *et al.* 2023; Yang, Wang *et al.* 2023). *Caenorhabditis elegans* has at least seven predicted lipocalins (LPR-1-LPR-7), only two of which have been studied to date. Their cargos are unknown but could include matrix-regulating hormones, lipids, or lipid-modified components of the aECM. LPR-1 is apically secreted from external epithelia but does not appear to incorporate into matrix (Pu *et al.* 2017). Nevertheless, loss of *lpr-1* phenocopies many precuticle mutants (Forman-Rubinsky *et al.* 2017). LPR-3 is a bona fide precuticle matrix component (Forman-Rubinsky *et al.* 2017).

#### Chondroitin proteoglycans

Chondroitin proteoglycans (CPGs) are a large group of unrelated proteins that are all post-translationally modified by a chondroitin glycosaminoglycan attachment. This group includes the precuticle ZP- and fibrillin-related protein FBN-1, two eggshell components (CPG-1 and CPG-2), and many other uncharacterized proteins (Olson *et al.* 2006; Noborn *et al.* 2018). Mutants lacking chondroitin have embryonic lethal and “squashed vulva” (Sqv) phenotypes that suggest additional CPGs fill the space between the early embryo and the eggshell and are part of the vulva lumen aECM (Herman *et al.* 1999; Hwang *et al.* 2003; Cohen, Sparacio, *et al.* 2020).

#### SPIA-1 and DPY-6 cysteine cradle family

These are six nematode-specific secreted proteins that all share a novel cysteine-cradle domain (CCD-aECM) at their C-termini, predicted to form a highly hydrophobic groove that can bind an α helix to make potential homo- or hetero-dimers (Sonntag *et al.* 2024). SPIA-1 (suppressor of persistant immune activation) was identified as a suppressor of the constitutive immune activation observed in furrow Dpy mutants. It is a precuticle component that localizes specifically to developing furrow regions (Sonntag *et al.* 2024). DPY-6 belongs to this family but also has a large mucin-like domain (Sun *et al.* 2022). This gene family exhibits a pattern of expression peaking at the core of every larval stage, synchronizing with the expression peaks of precuticle and furrow collagen genes, except for *dpy-6*, which peaks right at the onset of each cycle (Fig. 1c) (Meeuse *et al.* 2020; Sonntag *et al.* 2024).

#### Mucins

Mucins are serine- and threonine-rich, highly O-linked glycosylated, gel-forming components of mucus and other aECMs (Corfield 2015). *Caenorhabditis elegans* has >20 predicted mucins (Hansen *et al.* 1998), some of which contain both mucin-like and other matrix domains. DPY-6 is a mucin required for proper body and mouth shape in both *C. elegans* and *Pristionchus pacificus* and may be part of the cuticle or precuticle (Sun *et al.* 2022). Other mucins include the osmotic response regulator OSM-8 (Rohlfing *et al.* 2011), the copulatory plug protein PLG-1 (Palopoli *et al.* 2008), and the precuticle ZP protein LET-653 (Gill *et al.* 2016). Mucins are believed to be a major component of the outer surface coat of the cuticle (Parsons *et al.* 2014) (*The Cuticle*), though the specific mucins there are unknown.

#### Hedgehog-related proteins

Hedgehog (Hh) and Patched are an important ligand-receptor signaling pair in many animals, but Patched and its RND transporter relatives are also involved in transport of Hh and other lipophilic cargo across membranes (Nikaido 2018; Zhang and Beachy 2023). *Caenorhabditis elegans* lacks a true Hh as well as key downstream components of the Hh signaling pathway. However, *C. elegans* does have large families of divergent Hh-related (Hh-r) proteins (GRD/Groundhog, GRL/Groundhog-like, WRT/Warthog, QUA/Quahog), and Patched-related (PTR) proteins whose loss can cause defects in molting, pathogen sensitivity, epidermal responses, and precuticle and/or cuticle organization (Zugasti *et al.* 2005, 2016; Bürglin and Kuwabara 2006; Hao, Aspöck *et al*. 2006, Hao, Johnsen *et al.* 2006; Baker *et al.* 2021; Cohen *et al.* 2021; Zárate-Potes *et al.* 2022; Serra *et al.* 2024). Some of the Hh-r proteins appear to be actual structural components of the precuticle (GRL-7, GRL-18, WRT-10) and/or cuticle (GRL-2, GRL-18, QUA-1) based on their spatiotemporal localization patterns, and limited fluorescence recovery after photobleaching (FRAP) (Hao, Mukherjee *et al*. 2006; Liégeois *et al.* 2006; Chiyoda *et al.* 2021; Fung *et al.* 2023; Serra *et al.* 2024). Some PTR proteins may be involved in endocytosis or secretion of Hh-r proteins or other aECM components (Perens and Shaham 2005; Zugasti *et al.* 2005; Oikonomou *et al.* 2011; Rohlfing *et al.* 2011; Chiyoda *et al.* 2021; Cohen *et al.* 2021). Phylogenetic analyses suggest that these could be the most ancient roles of the Hh and PTR families (Bürglin 2008; Nikaido 2018; Roelink 2018). Some of the Hh-r and PTR proteins also appear to have signaling roles (Lin and Wang 2017; Kume *et al.* 2019; Templeman *et al.* 2020; Chiyoda *et al.* 2021; Wang *et al.* 2023), although their downstream targets are still unclear. Therefore, the aECM could serve as a reservoir of such signals (*Future prospects*).

## The precuticle

The precuticle is a recently discovered transient matrix that precedes (and is gradually replaced by) each new cuticle. The precuticle is present during embryo elongation and before molting during each larval stage, but it then disappears as the molt nears completion (Figs. 1 and 3d). As such, the precuticle is present during periods when epithelia undergo large changes in shape, and it is critical to resist or distribute morphogenetic forces to support epithelial shaping and integrity during these periods. The precuticle also influences subsequent cuticle structure and function, including the formation and shaping of cuticle alae ridges and the exchange of old and new cuticles during molting.

### The embryonic sheath and embryo elongation

**Fig. 5.**
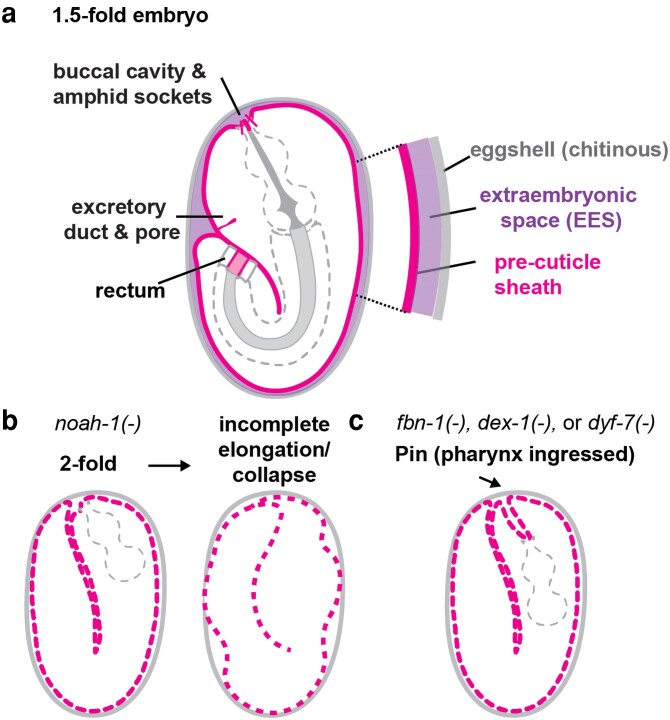
The embryonic sheath precuticle. a) Schematic of 1.5-fold embryo, with sheath (pink), extraembryonic space (purple), and eggshell (gray). At this stage, the extraembryonic space contains a mix of secreted precuticle and cuticle proteins that have not yet assembled (Birnbaum *et al.* 2023). Precuticle lined interfacial tubes are also shown. b) Defective elongation in a *noah-1* mutant (adapted from Vuong-Brender *et al.* (2017)). Dashed lines indicate abnormal sheath. c) Pharynx ingressed (Pin) phenotype observed with incomplete penetrance in *fbn-1*, *dex-1*, *and dyf-7* mutants. While FBN-1 is a component of the epidermal sheath (Kelley *et al.* 2015; Balasubramaniam *et al.* 2023), DEX-1 and DYF-7 contribute to buccal precuticle and amphid glia sensory matrices (Heiman and Shaham 2009; Cohen *et al.* 2019). It therefore appears that several different matrices contribute to buccal integrity.

The embryonic body precuticle, also known as the sheath (Fig. 5a), was observed by scanning electron microscopy as early as 1986, when it was presumed to be an initial layer of the L1 cuticle (Priess and Hirsh 1986; Costa *et al.* 1997). Its molecular contents and their transience only became recognized ∼30 years later.

NOAH-1, NOAH-2, FBN-1, LET-4, and EGG-6 are the earliest known markers of the sheath beginning before the 1.5-fold stage of embryogenesis (Mancuso *et al.* 2012; Kelley *et al.* 2015; Vuong-Brender *et al.* 2017; Balasubramaniam *et al.* 2023). Some other sheath components, such as LPR-3, WRT-10, GRL-7, and SPIA-1, join an hour or more later (Birnbaum *et al.* 2023; Serra *et al*. 2024; Sonntag *et al.* 2024). Interestingly, both GRL-7 and SPIA-1 are furrow-specific precuticle components (Chiyoda *et al.* 2021; Serra *et al*. 2024; Sonntag *et al.* 2024). Precuticle components remain in the matrix for only a few hours, until the completion of embryo elongation and the assembly of the cuticle, at which point they are removed by endocytosis (Birnbaum *et al.* 2023; Serra *et al*. 2024; Sonntag *et al.* 2024) (Fig. 1b). These genes will be reexpressed for another four times through the molting cycle (Fig. 1c) (Meeuse *et al.* 2020).

Some components of the sheath are essential for embryo elongation and were discovered in genetic screens for elongation-defective mutants. In the absence of *noah-1, noah-2*, or both *let-4* and *sym-1*, the embryo begins to elongate but then retracts and collapses before reaching its full length (Mancuso *et al.* 2012; Vuong-Brender *et al.* 2017) (Fig. 5b). Elongation defects are most severe when multiple sheath factors are depleted simultaneously or when the sheath is destroyed by trypsin treatment; in the most severe cases embryos do not elongate beyond the 2-fold stage (Priess and Hirsh 1986; Vuong-Brender *et al.* 2017). The sheath has been proposed to mechanically connect to epidermal HD structures that are also important for embryo elongation (Zhang *et al.* 2011; Vuong-Brender *et al.* 2017; Lardennois *et al.* 2019) (*The cuticle*).

### Precuticle in the buccal cavity

The buccal cavity or mouth of the worm consists of specialized epithelial cells that connect the most anterior epidermal cells to the pharynx or foregut (Thomson 1976). The buccal cavity is lined by a collagenous cuticle that appears continuous with that of the epidermis and differs considerably from the chitin-based cuticle of the pharynx (Wright and Thomson 1981; Zhang *et al.* 2005). In the embryo, this cavity is filled by several precuticle proteins, including DEX-1 (Cohen *et al.* 2019). Loss of FBN-1 or DEX-1 causes a “pharynx ingressed” (Pin) phenotype in which the anterior epidermis is pulled inward to the body during embryo elongation, reflecting changes in the mechanical properties of the epidermis or buccal cavity (Kelley *et al.* 2015; Cohen *et al.* 2019) (Fig. 5c). Loss of the amphid glia sensory matrix component DYF-7 can also cause a Pin phenotype (Cohen *et al.* 2019), consistent with the known mechanical connections between the anterior epidermis and glia (Heiman and Shaham 2009).

### Precuticle in the excretory duct and pore tubes

**Fig. 6.**
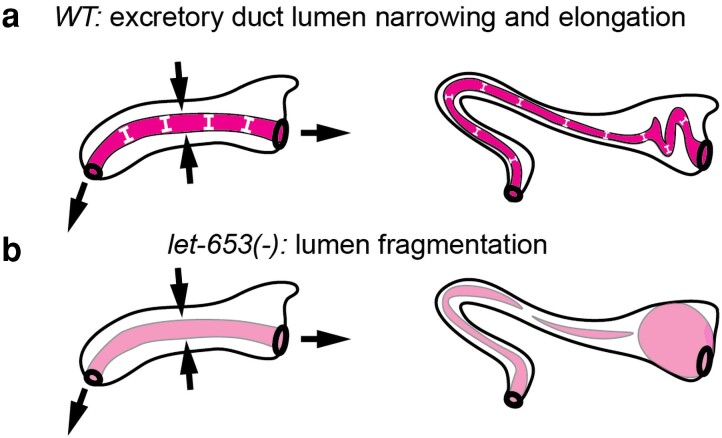
Excretory duct tube defects of precuticle mutants. a) the duct lumenal precuticle (pink) expands lumen diameter (white bars) and counters morphogenetic forces (arrows) involved in lumen elongation and narrowing. b) In *let-653(−)* or other mutants with an abnormal precuticle (light pink), the duct lumen fragments during elongation and fluid then accumulates in the “upstream” region nearest the canal cell. Adapted from Gill *et al.* (2016).

The excretory duct and pore are narrow single-celled tubes that connect the osmoregulatory excretory canal cell to the outside environment to allow for fluid excretion (Sundaram and Buechner 2016). Precuticle matrix fills and shapes these tubes during their morphogenesis, which occurs during embryo development (Gill *et al.* 2016) (Fig. 6). Several components of this matrix were initially identified through genetic screens for “rod-like” L1 lethal mutants with excretory defects (Mancuso *et al.* 2012; Gill *et al.* 2016; Forman-Rubinsky *et al.* 2017; Cohen *et al.* 2019). In these mutants, the duct and pore initially form tubes and begin to elongate, but their lumens become increasingly irregular in diameter and eventually lose patency, preventing fluid excretion (Fig. 6). Ectopic expression of one duct aECM component, the ZP and mucin protein LET-653, was able to expand the embryonic gut lumen, suggesting an intrinsic lumen-expanding activity (Gill *et al.* 2016).

### Precuticle in glial socket pore cells

Glial socket pore cells provide an opening to the external environment through which chemosensory neurons, such as the amphids and phasmids, can extend ciliated dendrites to send and receive chemical cues (Ward *et al.* 1975) (Fig. 3c). Glial pores form single-celled tubes that are sealed by apical junctions similar to those in epithelia, and they are lined by precuticle and eventually cuticle aECM (Low *et al.* 2019; Heiman 2022). Integrity of the amphid and phasmid pores can be assayed with dye-filling, since pore opening allows lipophilic dyes like DiI to enter the glial channel to stain the neurons within (Perkins *et al.* 1986). Mutations in some Patched-related proteins (DAF-6, CHE-14) and precuticle factors (LPR-1, LET-4) cause a dye-filling defective (Dyf) phenotype (Perens and Shaham 2005; Liégeois *et al.* 2007; Stone, Hall *et al.* 2009; Mancuso *et al.* 2012), suggesting that socket pore patency may require those factors.

Mechanosensory neurons such as CEP also have glial socket cells, but those sockets do not form an open pore and are instead covered by a sheet of cuticle in which the neuron endings are embedded (Ward *et al.* 1975). When the CEP-containing sensillum is remodeled to accommodate the male-specific CEM sensory neuron, the Hh-r protein GRL-18 forms a transient ring structure that marks the newly developing pore opening (Fung *et al.* 2023).

### Precuticle in the developing L4 vulva

**Fig. 7.**
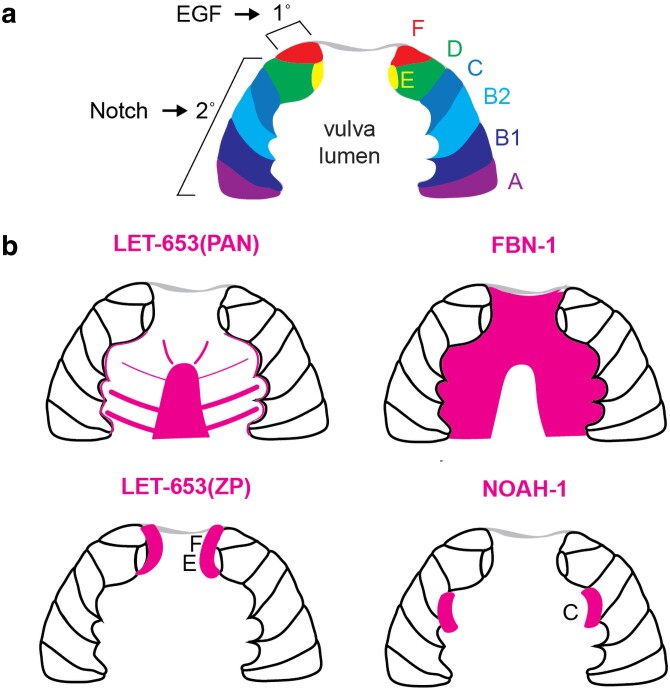
Precuticle ZP protein localization in the developing vulva lumen (mid-L4). a) EGF and Notch signaling promote 1˚ and 2˚ lineages that give rise to seven different vulva cell types (vulA-vulF) (Sharma-Kishore *et al.* 1999). b) The vulva lumen central cone matrix is marked by the plasminogen/apple-like (PAN) domains of LET-653, but mostly excludes FBN-1. The LET-653 ZP domain and NOAH-1 mark specific vulva cell surfaces (Gill *et al.* 2016; Cohen, Sparacio *et al.* 2020).

The vulva is a large multicellular tube that connects the uterus to the outside environment to allow egg-laying. It develops during the L3 and L4 larval stages as an inward invagination from the ventral epidermis and then becomes cuticle-lined in the adult (Sharma-Kishore *et al.* 1999). During L4 vulva morphogenesis, the vulva lumen is filled with precuticle matrix, and the large size of this lumen (∼10 microns in diameter) allows the complexity of the matrix to be visualized more easily than in most other tissues (Cohen, Sparacio *et al.* 2020). This vulva matrix consists of both membrane-associated components and a central cone-like structure that sits within the luminal cavity and connects to the membrane-associated matrix via fibrils that radiate outward from it (Fig. 7). Each vulva cell type appears to have a somewhat different precuticle aECM along its luminal membrane (Cohen, Sparacio *et al.* 2020) (Fig. 7), supporting the idea that aECM composition is one important aspect of specific epithelial cell identities.

The vulva lumen matrix is important to shape the developing vulva tube. Removal of a single precuticle matrix factor generally has modest effects, primarily altering the later steps of vulva eversion preceding the L4-adult molt (Cohen, Sparacio *et al.* 2020). Mutants lacking the enzymatic pathway for making the glycosaminoglycan chondroitin show a dramatic reduction in vulva lumen size, suggesting that CPGs help inflate the lumen (Herman *et al.* 1999; Hwang *et al.* 2003). On the other hand, combined depletion of CPGs and the ZP protein LET-653 leads to over-expansion of the dorsal vulva lumen, indicating more complex effects on cell shapes (Cohen, Sparacio *et al.* 2020). FBN-1 is one specific CPG involved in vulva shaping, but the others remain to be identified.

### Precuticle and alae formation

Alae are longitudinal ridges found in the cuticles of L1s, dauer larvae, and adults (Cox, Staprans *et al.* 1981) (Figs. 2b and 7a, b). They form specifically over the lateral seam epidermis and have very characteristic shapes that vary between the different life stages where they are found. Because alae are purely aECM structures, they raise the interesting question of how a complex matrix structure can be shaped outside of the cell.

**Fig. 8.**
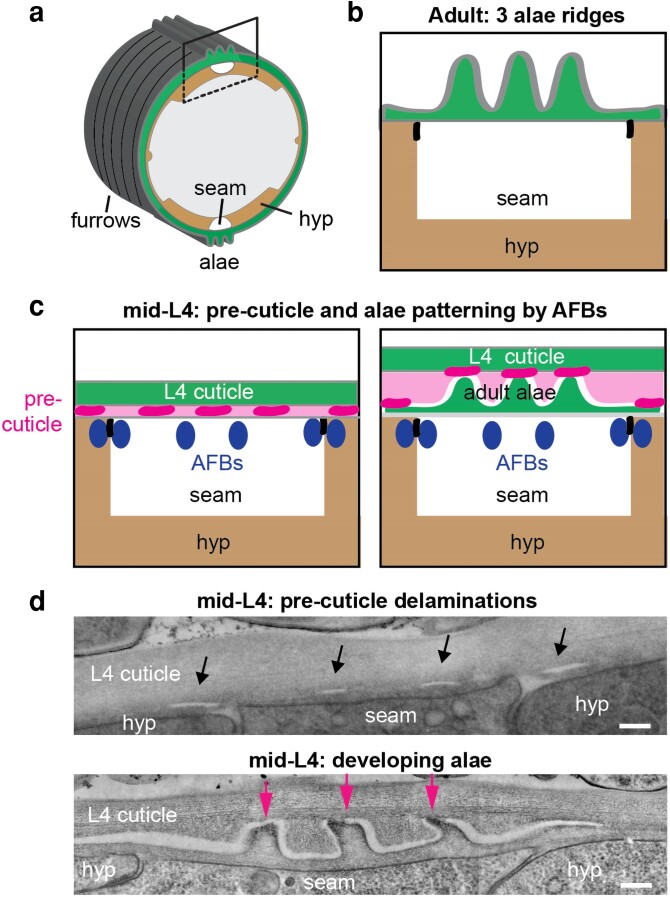
Adult alae form via AFB-dependent precuticle delamination. a) Schematic diagram of the adult worm; box indicates region of interest in b–d. b) Adult alae consist of three vertical cuticle ridges (green) above the seam. The seam is linked to the surrounding hyp7 syncytium by apical junctions (black). c) Arrangement of AFBs and precuticle factors as alae develop underneath the L4 cuticle (Katz *et al.* 2022). LPR-3 (magenta) marks future ridges, while LET-653 (light pink) marks future valleys. Cortical AFBs (blue) in the underlying seam and hyp7 also correspond to future locations of valleys. Left panel depicts an earlier stage (L4.4) corresponding to the top TEM image in (d), while right panel depicts a later stage (L4.6) when alae have begun to form. d) TEM images. In top image (L4.4), black arrows point to four small slits or delaminations in the precuticle underneath the L4 cuticle. These correlate with locations of AFBs, which are needed to pattern LPR-3 and the alae (Katz *et al.* 2022). In bottom image (L4.6), pink arrows point to newly formed alae tips, which are regions that have remained adherent to the L4 cuticle. Image credits: Ken Nguyen and David Hall.

Many precuticle mutants either lack adult alae ridges or have very disorganized and mis-shapen alae ridges (Forman-Rubinsky *et al.* 2017; Katz *et al.* 2022). A recent study of adult alae proposed that the precuticle matrix relays cytoskeletal patterns to the developing cuticle to form these ridges, and that the process involves matrix delamination (Katz *et al.* 2022) (Fig. 8). Interestingly, a reciprocal relationship between long actin filament bundles (AFBs) and aECM ridges has been observed in *C. elegans* adult alae, *Drosophila* bristles (Tilney *et al.* 1995), and butterfly wing scales (Dinwiddie *et al.* 2014), suggesting that shared mechanisms could be involved in patterning all these structures.

The L1 and dauer alae appear to form through a different mechanism that instead involves cuticle matrix constriction and buckling (Singh and Sulston 1978; Sapio *et al.* 2005), but these alae also require some precuticle factors such as LET-653 (Gill *et al.* 2016) in addition to a set of cuticle proteins (*The cuticle*).

### Precuticle and molting

Multiple epidermal precuticle proteins (NOAH-1, NOAH-2, FBN-1, and LPR-3) are required for successful molting (Frand *et al.* 2005; Forman-Rubinsky *et al.* 2017). After RNAi knockdown of these factors, larvae initiate molting but then fail to fully shed the old cuticle or lose tissue integrity during the molting process. This phenotype may reflect a general requirement for proper precuticle organization in order to separate the old and new cuticle matrices. Alternatively, it could reflect more specific roles for individual precuticle factors in positioning or activating relevant molting enzymes.

### Precuticle secretion, assembly/disassembly, and endocytosis

While precuticle dynamics suggest a series of highly regulated processes, little is known yet about the trafficking pathways used for precuticle secretion and endocytosis, or about the mechanisms that control precuticle matrix assembly and disassembly. RAB-11 is required for apical secretion of cortical granules containing CPGs (Sato *et al*. 2008) and likely promotes secretion of additional aECM factors, but specialized carriers such as Patched-related proteins and lipocalins could be involved in the transport of specific cargos (*Introduction*). VHA-5, a V0-a subunit of the vacuolar ATPase proton pump, is required for apical secretion of some Hh-r proteins through multivesicular bodies (Liégeois *et al*. 2006). The tetraspan proteins CUTI-1 and RDY-2 also have been implicated in matrix secretion and their loss causes defects reminiscent of some precuticle mutants (Liégeois *et al.* 2007; Fritz and Behm 2009).

Precuticle assembly occurs in precise temporal and spatial patterns. In the embryo, vulva, and alae regions, at least some precuticle proteins appear initially diffuse in the extracellular space before subsequently assembling into recognizable matrix structures, suggesting some delay in the assembly process (Cohen, Sparacio *et al.* 2020; Katz *et al.* 2022; Birnbaum *et al.* 2023). Some precuticle proteins coat the surface(s) of those cells in which they are produced (Fung *et al*. 2023; Serra *et al*. 2024), whereas ZP proteins appear to assemble on only a subset of their source cells or on different cells altogether (Heiman and Shaham 2009; Gill *et al.* 2016; Cohen, Sparacio *et al.* 2020). ZP protein assembly in many cases requires C-terminal proteolysis at a furin-like motif (Bokhove and Jovine 2018; Cohen, Bermudez *et al*. 2020), so it is possible that the expression or activity of relevant proteases is one controlling factor. Destination cells may also express specific membrane-anchored matrix organizers that then bind and recruit other factors (Cohen, Bermudez *et al*. 2020).

Precuticle disassembly presumably involves matrix proteases, but these have not been identified. In Drosophila, some transmembrane ZP proteins are released from cell surfaces via the action of Notopleural, a membrane-anchored trypsin family protease, which cleaves in between the ZPn and ZPc domains (Drees *et al*. 2019, 2023). *Caenorhabditis elegans* has 15 members of the trypsin protease family, but so far none have been implicated in precuticle or cuticle remodeling.

The precuticle is cleared by endocytosis, but what triggers it is unknown. Drosophila ZP proteins are endocytosed via a typical clathrin-mediated pathway (Drees *et al*. 2023). *Caenorhabditis elegans* NEKL kinases are required for molting and for clathrin-mediated endocytosis of some matrix-relevant cargo(s) (Joseph *et al*. 2020, 2023), so they are good candidates to be involved in precuticle endocytosis. Ultimately, most acid-tolerant mCherry fusions for precuticle proteins accumulate in lysosomes (Chiyoda *et al*. 2021; Birnbaum *et al*. 2023; Clancy *et al*. 2023), suggesting that endocytosed precuticle proteins are broken down in the lysosome and their components recycled. Indeed, epidermal lysosomes become highly active late in each larval stage, although this has been attributed to collagen turnover (Miao *et al.* 2020).

## The cuticle

The cuticle is the collagenous aECM structure present throughout each larval stage and in the adult. The epidermal cuticle serves as an exoskeleton for the attachment of body muscles to allow motility (Francis and Waterston 1991) and it shapes the body by resisting both muscle tone and hydrostatic pressure (Park *et al.* 2007; Petzold *et al.* 2011; Sanzeni *et al.* 2019). It protects the worm from environmental insults, including pathogens, toxins, and desiccation (Dodd *et al.* 2018; Martineau *et al.* 2021; Urso and Lamitina 2021), plays important roles in social interactions like mating (Garcia *et al.* 2001; Weng *et al.* 2023), transmits mechanosensory information to the nervous system (Sanzeni *et al.* 2019), and has even been proposed to serve an ear-like function by sensing airborn sound (Iliff *et al.* 2021).

Cuticle turnover during larval molts allows each stage to have a unique cuticle that is presumably tailored to its specific needs. For example, as described below, the cuticles of different life stages vary with respect to collagen composition (Table 1, Supplementary Table 1), number and appearance of ultrastructural layers (Cox, Staprans *et al.* 1981), presence of alae ridges, or attractiveness to mates (Weng *et al.* 2023). Molting also allows shedding of attached pathogens (Hodgkin 2019). Recently, a concerted effort to fluorescently tag and visualize endogenous cuticle collagens has provided a plethora of new tools to investigate their cell biology and regulation (Adams *et al.* 2023; Birnbaum *et al.* 2023).

**Table 1.** Examples of stage-specific body collagens.

Collagen	Stage*^a^*	References
BLI-1	Adult	(Adams *et al.* 2023)
BLI-2	Adult	(Adams *et al.* 2023)
BLI-6	Adult	(Adams *et al.* 2023)
COL-2	Dauer	(Kramer *et al.* 1985; Shih *et al.* 2019)
COL-7	Adult	(Liu *et al.* 1995; Jackson *et al.* 2014)
COL-19	Adult	(Liu *et al.* 1995; Thein *et al.* 2003)
COL-38	Adult	(Jackson *et al.* 2014; Abete-Luzi and Eisenmann 2018)
COL-40	Dauer	(Shih *et al.* 2019)
COL-49	Adult	(Jackson *et al.* 2014; Abete-Luzi and Eisenmann 2018)
COL-62	Adult	(Jackson *et al.* 2014; Taffoni *et al.* 2020)
COL-63	Adult	(Jackson *et al.* 2014; Abete-Luzi and Eisenmann 2018)
COL-183	Dauer	(Shih *et al.* 2019)
DPY-5	L2-Adult	(Jackson *et al.* 2014; Meeuse *et al.* 2020)
DPY-14	L1	(Gallo *et al.* 2006)
DPY-17	L1	(Jackson *et al.* 2014; Meeuse *et al.* 2020; Birnbaum *et al.* 2023)
ROL-6	L2-Adult	(Jackson *et al.* 2014; Meeuse *et al.* 2020; Aggad *et al.* 2023)

^*a*^Stage of cuticle incorporation, which typically is one stage after transcription is detected since the new cuticle is made prior to the molt (Fig. 1). However, some adult-specific collagens such as *col-62* are primarily transcribed in the adult.

### Cuticle composition differs between life stages and influences body shape and environmental and social interactions

*Caenorhabditis elegans* has at least 173 different cuticle collagens (Teuscher *et al*. 2019). While a majority are expressed cyclically at each life stage (Fig. 1c) (Hendriks *et al.* 2014; Meeuse *et al.* 2020), not all of these collagens are expressed in all stages or tissues. Indeed, RNA sequencing data from MODEncode and tissue specific targeted DamID suggest that stage-specific expression of collagens may be quite prevalent (Gerstein *et al.* 2010; Jackson *et al.* 2014; Katsanos *et al.* 2021). Stage-specific expression likely involves the heterochronic pathway acting with different terminal transcription factors (Liu *et al*. 1995; Rougvie and Moss 2013). At least one cuticle collagen gene, *col-182*, became a pseudogene during N2 lab strain domestication and its absence sensitizes N2 to the effects of other collagen mutations (Noble *et al.* 2020).

#### Larval cuticles maintain body shape

The first hints that collagen composition varies across stages came from the genetic analysis of collagen mutants. Many of these cause changes in body shape, posture, or cuticle appearance that are stage-specific (Brenner 1974; Wood 1988; Cho *et al.* 2021; Chandler and Choe 2022; Mendoza *et al.* 2022). For example, *dpy-17* mutants are very short and fat (Dumpy, Dpy) at hatch, but less Dpy as adults, whereas *dpy-5* mutants show the opposite pattern. Many Roller (Rol) mutants begin to roll only at late larval stages or in the adult. *bli-1, bli-2,* or *bli-6* mutants appear normal until the adult stage, when they begin to blister (Bli). These observations can be explained by the fact that these collagens are only incorporated into the body cuticles of a subset of life stages (Table 1).

L1 cuticle collagens are important to maintain body shape after initial embryo elongation and clearance of the precuticle sheath (*The precuticle*). The only known essential cuticle collagens are SQT-3 and DPY-14 (van der Keyl *et al.* 1994; Gallo *et al.* 2006). *sqt-3* mutants initially elongate but then retract and arrest as collapsed embryos (Priess and Hirsh 1986). DPY-17 is an important functional partner of SQT-3 and is required for efficient SQT-3 secretion and L1 body shape (Novelli *et al.* 2006; Birnbaum *et al.* 2023). Although SQT-3 is a component of cuticle at every stage, we observed that DPY-17 is only present in early larvae (Table 1), suggesting other collagens may partner with SQT-3 at later stages. Several collagens important for adult body shape, including DPY-5, are not present in the L1 cuticle but appear at later stages (Table 1). LON-3, which is present at all stages, is the only known cuticle collagen whose loss leads to a longer body shape (Lon phenotype) (Nyström *et al.* 2002).

#### Dauer cuticle confers stress-resistance

Many changes to cuticle structure must occur in dauer larvae, which have a very narrow body shape and show extreme resistance to detergents and other environmental insults (Cassada and Russell 1975; Androwski *et al.* 2017). By electron microscopy, the dauer cuticle appears thicker than that of other larval stages and its basal zone has a unique woven appearance (Cox, Staprans *et al.* 1981). It also contains more insoluble materials than the cuticles of other stages. Furthermore, a dauer-specific matrix plug blocks the buccal cavity to prevent feeding (Wolkow and Hall 2011). Therefore, matrix and matrix remodeling factors must be key downstream targets of the known dauer-controlling signaling pathways (Gumienny and Savage-Dunn 2013; Baugh and Hu 2020). The gene *dex-1* is one such target (Flatt *et al.* 2019), and cuticlin genes important for dauer alae are also likely to be (Muriel *et al.* 2003; Sapio *et al.* 2005), along with dauer-specific collagen genes such as *col-2, col-40*, and *col-183* (Table 1) (Kramer *et al.* 1985; Jackson *et al.* 2014; Lee *et al.* 2017; Shih *et al.* 2019).

#### Adult cuticle contains unique collagens and social cues

Many collagens, including COL-7, COL-19, and COL-62, appear unique to the adult cuticle (Liu *et al.* 1995; Thein *et al.* 2003; Jackson *et al.* 2014; Abete-Luzi and Eisenmann 2018; Taffoni *et al.* 2020) (Table 1). The three known BLI collagens (BLI-1, BLI-2, BLI-6) are also present only in the adult, where they contribute to adult-specific cuticle struts (Adams *et al.* 2023) (see below). The heterochronic pathway and the terminal transcription factor LIN-29 promote the L4-to-adult transition and adult-specific collagen expression (Liu *et al.* 1995; Rougvie and Ambros 1995; Rougvie and Moss 2013; Abete-Luzi and Eisenmann 2018).

Interestingly, males can recognize the difference between the cuticles of adult hermaphrodites vs juveniles, males, or nematodes from distant species, even when these cuticles are purified away from the rest of the worm (Weng *et al.* 2023). Therefore, adult cuticles appear to contain important social cues that guide mating behavior. Several adult male-specific copulatory structures, such as the hook and spicules, are also made of cuticle and may contain unique contents (see below) (Sulston *et al.* 1980; Emmons 2005).

In the adult, many cuticle collagen genes continue to be expressed and can be further upregulated under certain mutant or stress conditions (Pujol, Zugasti *et al.* 2008; Jackson *et al.* 2014; Ewald *et al.* 2015; Palani *et al.* 2023). Indeed, continuous cuticle expansion and repair does occur to accommodate adult growth, even though molting has ceased (Teuscher *et al.* 2024). The quality of the adult cuticle is a major determinant of healthy aging (Wolkow *et al.* 2017; Reich and Savage-Dunn 2023).

#### TGFbeta signaling and collagens

In both *C. elegans* and mammals, there are many intriguing links between TGFbeta signaling and extracellular matrix organization (Kim *et al.* 2018; Goodman and Savage-Dunn 2022). Two different *C. elegans* TGFbeta signaling pathways, involving the Activin-like ligand DAF-7 and the BMP-like ligand DBL-1, inhibit dauer entry and promote normal body size, respectively (Gumienny and Savage-Dunn 2013). Mutants in the *daf-7* pathway are dauer-constitutive (Daf-c) or dauer defective (Daf-d), while mutants in the *dbl-1* pathway are noticeably smaller (Sma) or longer (Lon) than wild-type. The DAF-7 pathway must ultimately repress the expression of dauer cuticle factors (see above). The DBL-1 pathway alters body size and cuticle organization via both positive and negative effects on collagen gene expression (Suzuki *et al.* 2002; Schultz *et al.* 2014; Madaan *et al.* 2018; Lakdawala *et al.* 2019). Cuticle collagen mutations also change the levels of *dbl-1* expression, suggesting a potential mechanosensitive feedback loop (Lakdawala *et al.* 2019; Madaan *et al.* 2020; Goodman and Savage-Dunn 2022).

### Cuticle composition differs between tissues

#### Seam epidermal cuticle differs from hyp7 cuticle

Consistent with the presence of different cuticle substructures over different epidermal cells (Fig. 2, see below), tissue specific targeted DamID revealed that some collagens are differentially expressed in the seam vs hyp7 (Katsanos *et al.* 2021). Less is known about the properties of cuticle over other hyp cells at the nose and tail (see Fig. 3a).

#### Interfacial tubes have unique cuticles

The cuticles of interfacial tubes differ from that of the epidermis in having no furrows or struts and fewer distinct ultrastructural layers (see below), although the cuticle of the buccal cavity and excretory pore do have distinctive striations or orbital ridges (Wright and Thomson 1981; Hall and Altun 2008). Imaging of collagen fusions has revealed that the excretory duct and pore and the vulva do not contain several of the body cuticle collagens examined to date (Sundaram lab, unpublished), but the vulva does contain BLI-6 (Adams *et al.* 2023). On the other hand, some collagen genes (*col-53, col-56, col-177*) and also some Hh-r proteins such as GRL-2 and GRL-18 are found only in specific interfacial tube cells (Fung *et al.* 2020, 2023; Serra *et al*. 2024) (Supplementary Table 1). An important question is what specific properties this diversity brings.

#### Male-specific cuticle structures

**Fig. 9.**
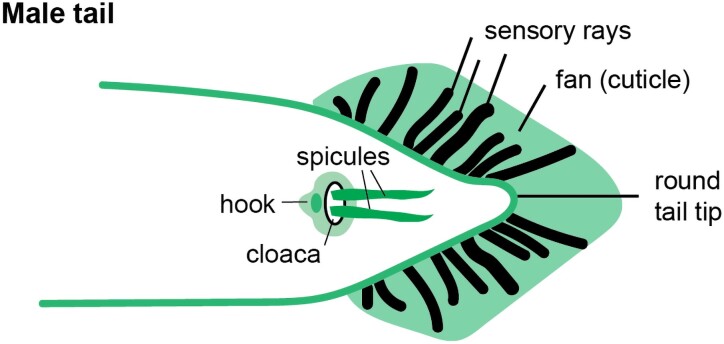
The adult male tail ventral view, showing cuticular structures (green) including the tail fan and the hook and spicules. The gubernaculum (not shown) is located on the dorsal side. Drawing based on Jiang and Sternberg (1999) and Lints and Hall (2009).

*Caenorhabditis elegans* adult males have an elaborate tail structure specialized for mating, with a cuticular fan and numerous male-specific sensilla (Sulston *et al.* 1980; Emmons 2005) (Fig. 9). Glial socket cells within some of these sensilla (the hook, spicules, and gubernaculum) produce highly refractive and hardened cuticle structures involved in vulva location, opening, and insemination (Sulston *et al.* 1980; Jiang and Sternberg 1999; Garcia *et al.* 2001). In the case of the spicules, socket glia withdraw after generating this cuticle, leaving the protruding spicules behind (Sulston *et al.* 1980).

Male tail morphogenesis also involves changing interactions between posterior epidermal cells and aECM. The tail fan is composed of acellular cuticle matrix, left behind after hyp(8–10) fusion and retraction at the L4-adult molt (Sulston *et al.* 1980; Nguyen *et al.* 1999; Kiontke *et al.* 2024). Ray glial cells and their associated sensory neurons extend across the fan because they (and thin protrusions of epidermis associated with them) remain linked to the adult cuticle after hyp(8–11) retraction, similar to what has been described for amphid sensory neurons and glia during embryogenesis (Heiman and Shaham 2009). Tip retraction also leads to the rounded shape of the adult male tail, rather than the tapered (“leptoderan”) shape of the juvenile or hermaphrodite tail or of the male tail in some other nematode species (Nguyen *et al.* 1999; Del Rio-Albrechtsen *et al.* 2006).

Cuticle and precuticle matrix dynamics during male tail development have not been characterized in detail yet, but several *ram* (ray morphology abnormal) or *mab* (male abnormal) mutants correspond to ZP proteins, collagens or collagen-modifying enzymes, and other putative aECM components (Baird and Emmons 1990; Yu *et al.* 2000; Tsang *et al.* 2007) (Supplementary Table 1). The *lep* (leptoderan) mutants define various regulators and targets of a heterochronic pathway that promotes the juvenile to adult transition (Del Rio-Albrechtsen *et al.* 2006; Mason *et al.* 2008; Nelson *et al.* 2011; Kiontke *et al.* 2019).

### Furrows and furrow collagens

**Fig. 10.**
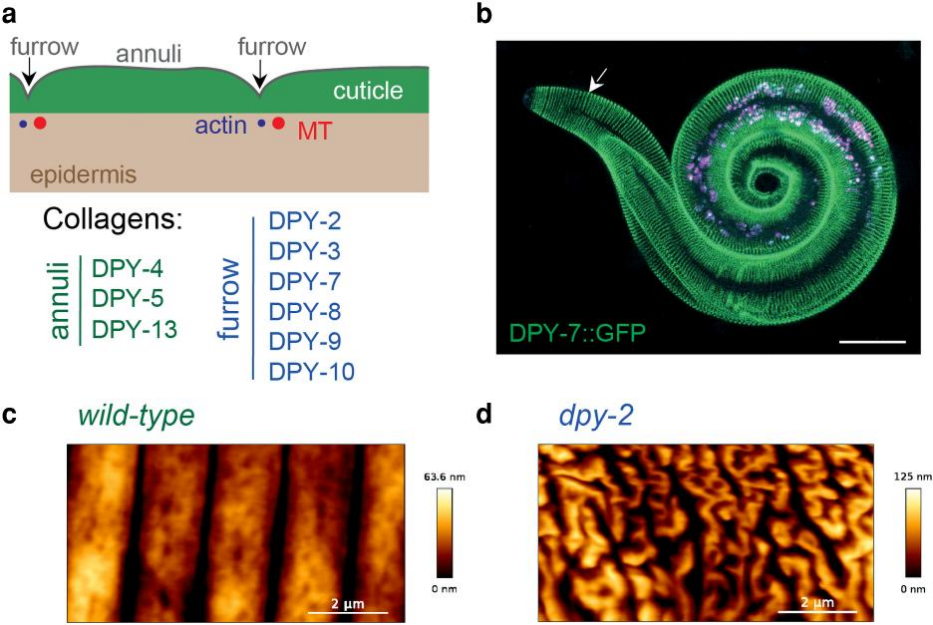
Furrows. a) The cuticle is decorated with periodic circumferential indentations called furrows, separating annuli. Several collagens mark specifically each structure. Actin and microtubules (MT) align transiently with furrows before each molt in the main lateral epidermis. b) A DPY-7::SfGFP L2 larvae is molting, detaching from its old L1 cuticle (arrow), scale bar 10 µm. Image credit: N.P. c) and d) are AFM topography of the cuticle of wild-type (c) and furrow-less *dpy-2* mutants (d) (from Aggad *et al.* (2023)).

At all stages, the body cuticle is organized into a series of circumferential bands, the annuli, which are each ∼1–1.5 microns wide and are separated by indentations called furrows (Figs. 2 and 10a, b) (Cox, Kusch *et al.* 1981, Cox, Staprans *et al.* 1981). Furrows and annuli develop in the first cuticle built during embryogenesis, and they increase in number and/or size in subsequent stages. These different substructures of the epidermal cuticle differ in content. For example, DPY-13 marks only annuli, whereas six collagens (DPY-2, −3, −7, −8, −9, and −10), named furrow collagens, localize specifically to cuticle furrows and their absence leads to furrow-less worms (McMahon *et al.* 2003; Dodd *et al.* 2018; Aggad *et al.* 2023) (Fig. 10). Genes encoding collagens and precuticle components cycle to generate the new cuticle with each molt. Furrow collagens are the first collagens to undergo cycling, indicating their early role in organizing the formation of the new matrix (Fig. 1c) (Meeuse *et al.* 2020; Adams *et al.* 2023; Sonntag *et al.* 2024).

#### Furrows monitor cuticle damage

Importantly, specific furrow loss in furrow-less mutants leads to the induction in the epidermis of an innate immune response with the expression of antimicrobial peptides, similar to the response to epidermal fungal infection or damages (Pujol, Cypowyj *et al.* 2008; Pujol N, Zugasti 2008; Zugasti *et al.* 2014; Dodd *et al.* 2018; Taffoni *et al.* 2020; Martineau *et al.* 2021). Additionally, an osmotic response is activated with a high expression of glycerol, in parallel to a detoxification response (Lamitina *et al.* 2006; Wheeler and Thomas 2006; Dodd *et al.* 2018; Chandler and Choe 2022). Although all three responses are started by the absence of furrows, they differ in their signaling and effectors. The innate response is partially relayed by the DCAR-1/GPCR receptor, p38/PMK-1 and STA-2/STAT transcription factor (Dierking *et al.* 2011; Zugasti *et al.* 2014; Dodd *et al.* 2018), while the osmotic response is controled by the hedgehog receptor PTR-23, the DRL-1 kinase and ELT-3/GATA, and the detoxification by SKN-1/Nrf (Rohlfing *et al.* 2011; Dodd *et al.* 2018; Wimberly and Choe 2022). This led to the suggestion that furrows define a common sensor lying in the cuticle that is activated by damage and translates complementary cellular responses in the epidermis (Dodd *et al.* 2018; Aggad *et al.* 2023). Furrow-less mutant also have an altered cuticle barrier function (Sandhu *et al.* 2021) that might be due to mispatterning of precuticle components (Sonntag *et al.* 2024) and an altered apical membrane organization and integrity (Wang *et al.* 2020; Aggad *et al.* 2023).

#### Furrows and body stiffness

Interestingly, furrow-less worms were found to have a reduced aECM stiffness by atomic force microscopy (AFM), and it is tempting to propose that aECM mechanical properties could be read by the epidermis to signal damage and induce appropriate responses (Aggad *et al.* 2023). Rather counter-intuitively, biophysical measurements with greater indentations probing the whole body revealed on the contrary that furrow-less mutants are twice as stiff as the wild type (Fechner *et al*. 2018). It is hypothesized that this increased body stiffness is related to an increase in hydrostatic pressure in the furrow-less mutants, probably due to their high glycerol content (Fechner *et al.* 2018) (Sonntag and NP, personal communication). Body stiffness also is used by males to control mating (Aggad *et al.* 2023; Weng *et al.* 2023). Furrow collagens were recently found to be positioned orthogonally to the cuticle in little posts aligned along the worm circumference (Adams *et al.* 2023). How these structures could change the mechanical properties of the cuticle and how it would be sensed by the epidemis is an open question.

#### Circumferential actin filament bundles underlie new cuticle furrows

During periods of initial cuticle synthesis in the embryo and larvae, circumferential AFBs sit directly underneath the developing cuticle furrows before being disassembled at hatch or following the molt (Priess and Hirsh 1986; Costa *et al.* 1997; Liégeois *et al.* 2007; Katz *et al.* 2022; Aggad *et al.* 2023). Although AFBs initially were proposed to pattern the furrows (Priess and Hirsh 1986; Costa *et al.* 1997), knockdown of actin in larvae did not perturb furrow organization (Katz *et al.* 2018). Instead, removal of furrow cuticle collagens prevented alignment of AFBs and adjacent microtubules during the late L4 in the lateral epidermis, indicating that matrix patterns the cytoskeleton (Aggad *et al.* 2023). The mechanical link between the furrow matrix and the AFBs that lead to the observed cycles of AFB assembly and disassembly is still to be discovered.

### Longitudinal alae components

During L1, dauer, and adult stages, the circumferential annular pattern is interrupted over the lateral seam epidermis, which instead contains longitudinal alae ridges (Cox, Staprans *et al.* 1981) (Figs. 2b and 7). The number and shape of alae ridges varies among the stages that have them. Seam cells that will secrete alae upregulate the chaperone BiP/HSP-4 in what appears to be a case of developmentally programmed anticipation of increased secretory load (Zha *et al.* 2019). The purpose of alae is not known but one speculated function is to provide traction for motility; if so, it is unclear why only certain stages would have them.

#### L1 and dauer alae

Collagens specific to the L1 and dauer alae are not yet known, but DEX-1 and different sets of cuticlin ZP proteins are required to form alae at different stages—CUT-3 and CUT-5 for L1 alae, and CUT-1 and CUT-5 for dauer alae (Sapio *et al.* 2005; Cohen *et al.* 2019; Flatt *et al.* 2019) (Table 2). CUT-6 also influences dauer alae shape and the relationship of alae and furrows (Muriel *et al.* 2003). CUT-1 is part of the seam cuticle underneath the alae based on immunostaining (Ristoratore *et al.* 1994), while CUT-6 localizes to longitudinal bands on the hyp7 side of the seam-hyp7 borders (Muriel *et al.* 2003). The seam narrows prior to alae formation and *cut* mutants that lack alae also have widened body shapes, leading (Sapio *et al.* 2005) to propose a ZP matrix buckling model in which biochemical compaction of more basal aECM layers could lead to seam narrowing and to buckling of more apical matrix layers above to form the alae.

**Table 2.** Contents of cuticle substructures.

Substructure	Components	References
Furrows	DPY-2, DPY-3, DPY-7, DPY-8*^a^*, DPY-9*^a^*, DPY-10, BLI-6*^b^*	(McMahon *et al.* 2003; Jackson *et al.* 2014; Zhu *et al.* 2023; Adams *et al.* 2023)
Alae (L1)	DEX-1, CUT-3*^a^*, CUT-5*^a^*, EPIC-1, EPIC-2	(Sapio *et al.* 2005; Cohen *et al.* 2019; Pooranachithra *et al.* 2024)
Alae (Dauer)	DEX-1, CUT-1, CUT-5*^a^*, EPIC-1, EPIC-3	(Sapio *et al.* 2005; Flatt *et al.* 2019; Pooranachithra *et al.* 2024)
Alae (Adult)	BLI-6, COL-12, EPIC-1, EPIC-2	(Chen *et al.* 2023; Adams *et al.* 2023; Pooranachithra *et al.* 2024)
Struts (Adult)	BLI-1, BLI-2, BLI-6, EPIC-1, EPIC-2	(Adams *et al.* 2023; Pooranachithra *et al.* 2024)

^*a*^DPY-8, DPY-9, CUT-3, and CUT-5 proteins have not been visualized but are presumed to be present at furrows (DPY-8 and DPY-9) or alae at specific stages (CUT-3 and CUT-5) based on transcriptional data and/or mutant phenotypes.

^*b*^BLI-6 is only present in adult.

#### Adult alae

The collagens BLI-6 and COL-12 and the epicuticlins EPIC-1 and EPIC-3 mark adult alae ridges (Adams *et al.* 2023; Chen *et al.* 2023; Pooranachithra *et al.* 2024) (Table 2). Interestingly, COL-12 marks alae even when its expression is driven with a hyp7-specific promoter (Chen *et al.* 2023), suggesting some alae components may derive from hyp7; this observation is consistent with imaging data showing transient seam narrowing and hyp7 apical expansion during early stages of alae formation (Katz *et al.* 2022).

During adult alae development, transient longitudinal AFBs form on each side of the seam-hyp7 border and also within the seam beneath what will eventually become the valleys between alae ridges (Katz *et al.* 2022; Aggad *et al.* 2023). These AFBs are important to pattern the transient precuticle, and ultimately the cuticle, into coherent alae ridge structures (Katz *et al.* 2022) (*The precuticle*) (Fig. 8). Longitudinal AFBs have not been observed in the hyp or seam at earlier larval stages, when seam cells have not yet fused and are still mitotically active. The cues that cause lateral AFBs to reorient longitudinally specifically at the L4 stage remain mysterious.

### Ultrastructural layers of the cuticle and their functions

**Fig. 11.**
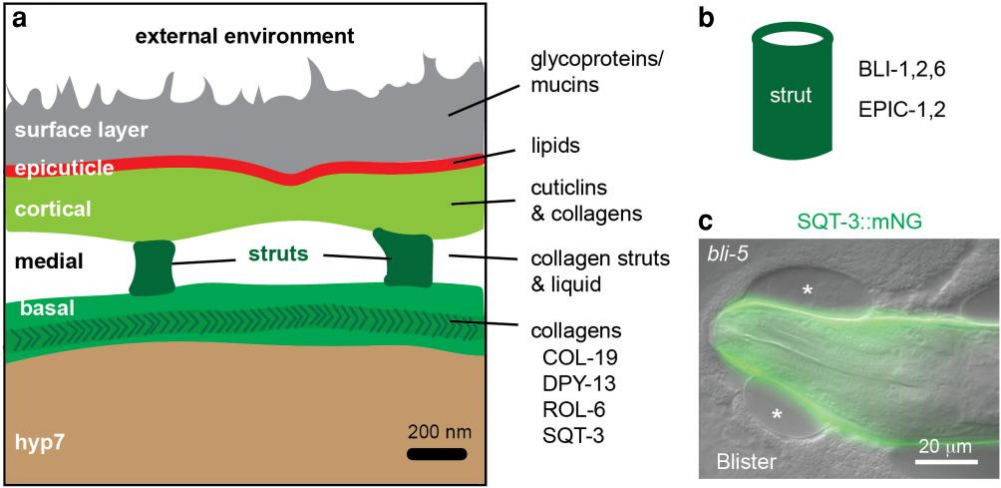
Adult cuticle layers and struts. a) Ultrastructural layer appearance, adapted from Adams *et al.* (2023). Some known contents are indicated at right; see text for details. b) Strut. BLI collagens define hollow cylinders by superresolution microscopy, suggesting other contents may fill the strut interior (Adams *et al.* 2023). Epicuticlins also localize to struts as well as to a cortical or epicuticle layer above them (Pooranachithra *et al.* 2024). c) Blistered mutants facilitate assessment of cuticle layer contents, since cortical and basal layers become widely separated as the medial layer expands (Adams *et al.* 2023). In this case, SQT-3::mNG is clearly in a basal cuticle layer below the blisters (asterisks). Image credit: M.V.S.

Early electron microscopy studies revealed that the body cuticle is organized into discrete ultrastructural layers (Fig. 2b), the number and appearance of which differ according to life stage (Cox, Kusch *et al.* 1981; Cox, Staprans 1981; Lints and Hall 2009). Each layer appears to have different molecular contents (Fig. 11a), although these are only partly defined.

#### Basal layer(s)

These are the deepest layers, adjacent to the plasma membrane, and consist mainly of collagens based on their degradation with collagenase (Cox, Kusch *et al.* 1981; Cox, Staprans 1981; Blaxter 1993). These layers vary noticeably among different stages. L1 larvae have just one basal layer, which contains short vertical striations; *sqt-3* collagen mutants perturb this striated layer, suggesting its importance for body shaping (Priess and Hirsh 1986). Older larvae and adults have at least two or three distinguishable basal layers, some of which appear fibrous by electron microscopy (Cox, Staprans *et al.* 1981; Peixoto and De Souza 1995). In addition to SQT-3 (Fig. 11c), ROL-6, DPY-13, and COL-19 collagens localize to these layers (Adams *et al.* 2023; Aggad *et al.* 2023). The opposite helical orientations of collagen fibers in different basal layers have been proposed to explain the left vs right Roller phenotypes of several collagen mutants (Peixoto *et al.* 1997; Bergmann *et al.* 1998).

#### Cortical layer(s)

These also contain collagens but also cuticlins, including CUT-1 and CUT-2 (Cox, Kusch *et al.* 1981; Cox, Staprans 1981; Blaxter 1993; Lassandro *et al.* 1994; Ristoratore *et al.* 1994). Enzymatic digestion suggested that the insoluble cuticlins occupy the most external cortical region (Cox, Kusch *et al.* 1981). By electron microscopy, the cortical region is fairly featureless and uniform in appearance across life stages, except under alae, where additional fibrillar material is present (Cox, Staprans *et al.* 1981). Several cuticlins are important for shaping the body and alae (Muriel *et al.* 2003; Sapio *et al.* 2005) (see above). A recent study of *C. elegans* epicuticlins EPIC-1–3 demonstrated a role in wound healing and localization to an outer cuticle layer (Pooranachithra *et al.* 2024).

#### The adult medial zone and struts

In adults, the basal and cortical layers are separated by an apparently fluid filled space called the medial zone. The lipid transfer protein GMAP-1 accumulates in this space and is required for its proper formation (Njume *et al.* 2022). Collagenous pillars or struts extend ∼200 nm across the medial zone to link the basal and cortical layers. The struts contain collagens BLI-1, BLI-2, and BLI-6, of which BLI-1 is unusually large and so may have relevant special attributes (Adams *et al.* 2023). In the absence of BLI-1 or BLI-2, struts are missing and the basal and cortical cuticle layers delaminate, leading to the blistered phenotype (Adams *et al.* 2023; de Melo *et al.* 2003). Recent super resolution analyses revealed that BLI collagens are assembled in hollow cylinders orthogonal to the surface of the cuticle (Fig. 11b) and are arranged in three circumferential rows, with two being adjacent to the furrow posts (Adams *et al.* 2023). EPIC-1 and EPIC-2 also localize to the struts but are not required for strut formation (Pooranachithra *et al.* 2024).

#### The epicuticle

The epicuticle is a thin electron-dense upper layer that contains many lipids and likely corresponds to the cuticle's permeability barrier, which can be tested by the exclusion of Hoechst dye (Moribe *et al.* 2004). This layer can be visualized with lipophilic dyes by both light and electron microscopy (Blaxter *et al.* 1992; Schultz and Gumienny 2012; Schultz *et al.* 2014; Pooranachithra *et al.* 2024). Biochemical analysis suggested that it contains a complex mix of fatty acids, phospholipids, triglycerides, and other lipids (Blaxter 1993). The GMAP-1 lipid transfer protein is required for proper cuticle barrier function and may help deliver phospholipids to this developing layer (Njume *et al.* 2022). Barrier function (and therefore potentially epicuticle secretion or assembly) also requires TGFbeta signaling (Schultz *et al.* 2014), furrow and strut cuticle collagens (Jackson *et al.* 2014; Sandhu *et al.* 2021) and a subset of precuticle proteins (LPR-3, LET-4, EGG-6) (Mancuso *et al.* 2012; Forman-Rubinsky *et al.* 2017). By analogy to *Ascaris suum*, the epicuticle may also contain epicuticlins (Bisoffi *et al.* 1996; Betschart *et al.* 2022; Pooranachithra *et al.* 2024).

#### The surface coat or glycocalyx

The surface coat is a carbohydrate-rich layer on the outermost cuticle surface that affects lectin binding patterns and sensitivity to bacterial and fungal pathogens (Link *et al.* 1992; Silverman *et al.* 1997; Gravato-Nobre *et al.* 2005; Novelli *et al.* 2009; Rouger *et al.* 2014; Zugasti *et al.* 2016). It likely contains mucins and other highly glycosylated proteins (Parsons *et al.* 2014), along with galactofuranose and rhamnose, two carbohydrates that are more typically found in microbes and appear to have arisen in nematodes via horizontal gene transfer (Novelli *et al.* 2009; Feng *et al.* 2016; Pandey *et al.* 2023). The surface coat is altered in *bus* (bacterially unswollen), *bah* (biofilm absent on head), and *srf* (surface abnormal) mutants, many of which encode glycosylation enzymes or transporters of nucleotide sugar (Höflich *et al.* 2004; Gravato-Nobre *et al.* 2005, 2011; Darby *et al.* 2007; Palaima *et al.* 2010; O’Rourke *et al.* 2023) (Supplementary Table 2). These genes are expressed primarily in the seam epidermis, suggesting that the seam may be responsible for producing the surface coat (Gravato-Nobre *et al.* 2011; O’Rourke *et al.* 2023).

Interestingly, surface mutants reduce the adhesion of the bacterial pathogen *Microbacterium nematophilum* to the rectum cuticle or *Yersinia pseudotuberculosis* to the head and therefore confer resistance to infection (Darby *et al.* 2007; O’Rourke *et al.* 2023). But they provoke the opposite phenotype, of increased adhesion and susceptibility during infection, with another cuticle-adhering bacteria of the genus Leucobacter (Hodgkin *et al.* 2013; O’Rourke *et al.* 2023), or during infection with the fungus *Drechmeria coniospora,* where spores also adhere to the cuticle with highest affinity to the vulva and other tube openings and the alae (Rouger *et al.* 2014; Zugasti *et al.* 2016). Therefore, the arrangement of the outer layer of the cuticle seems to have a restricted range of possible variations because of the equilibrium between advantageous and harmful relationships with microorganisms.

### Models for cuticle layer formation

The complex organization of the cuticle raises the interesting question of how its different layers and structures are built in the extracellular environment. A long-standing hypothesis has been that cuticle layers assemble through sequential waves of matrix secretion, with each wave of secreted matrix immediately assembling into a new layer and pushing the prior layer(s) outward (Johnstone and Barry 1996; Costa *et al.* 1997; Johnstone 2000). More recent observations require a revision of this model. First, the known components of the embryonic sheath/precuticle are removed by endocytosis rather than remaining as part of an outer cuticle layer (Vuong-Brender *et al.* 2017; Birnbaum *et al.* 2023). Second, since the surface coat appears to be made in the seam cells (Gravato-Nobre *et al.* 2011; O’Rourke *et al.* 2023), its contents most likely spread from there over the cuticle surface. Third, TEM suggests that the adult struts in the medial layer form after some of the adjacent basal and cortical layers (Adams *et al.* 2023). Finally, endogenously tagged fusions revealed that SQT-3 and DPY-17 basal layer cuticle collagens are secreted in soluble form into the extraembryonic space and remain there for several hours before they begin to assemble into the first cuticle matrix (Birnbaum *et al.* 2023). These collagen fusions accumulate outside of the precuticle layer (Fig. 1b), suggesting that collagens may pass through the precuticle or may actually assemble within it or on its outer surface (*Future Prospects*). Super-resolution and time-lapse imaging studies will be needed to better understand the relationship between these different matrices and layers.

The observed phased expression of different collagens and other matrix factors (Fig. 1c), while not sufficient to explain cuticle layer formation, likely facilitates it by ensuring that partners are present together at an appropriate time to form various layers and structures. Some collagens that function together may be cotranslationally routed to the ER, where they can begin to assemble into higher-order structures even prior to secretion (Novelli *et al.* 2006) (*Cuticle collagen maturation*).

### Connecting the cuticle to the epidermis

**Fig. 12.**
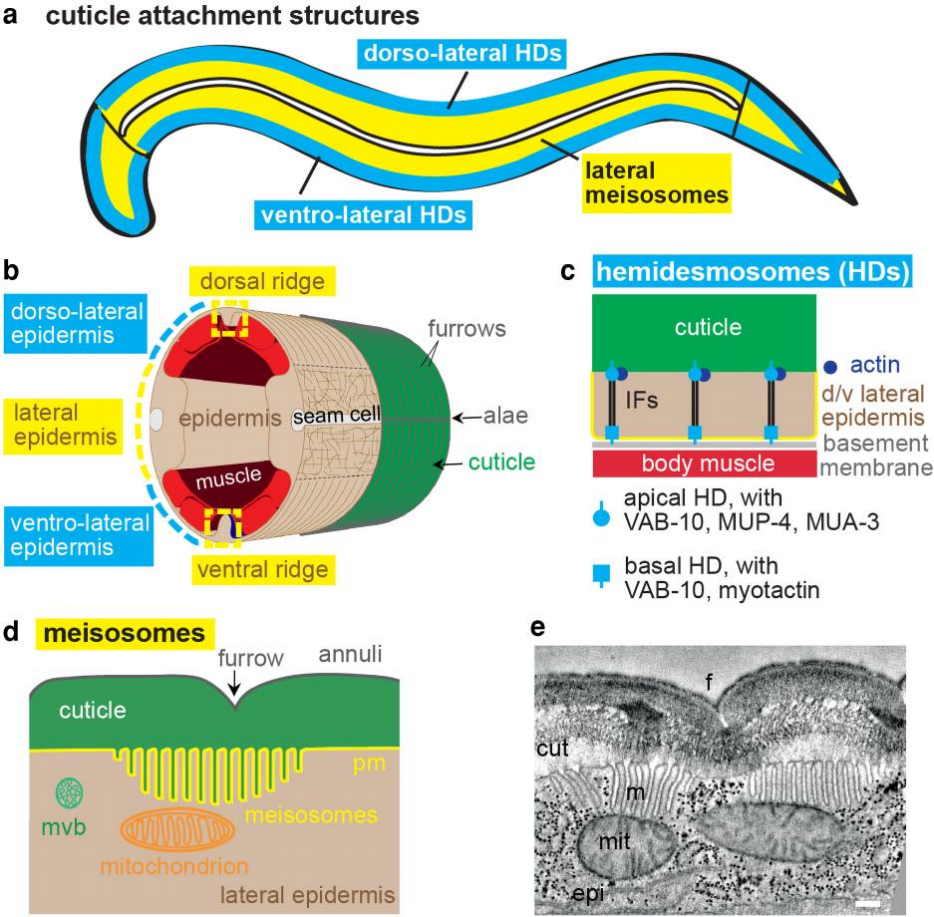
Cuticle attachment structures: HDs and meisosomes. a) The epidermal syncytium can be separated into two structurally different regions. The first one is dorso- and ventro-lateral, above the muscles where the epidermis is really thin and rich in hemidesmosomes (blue). The second includes the lateral epidermis and the ventral and dorsal ridges; it contains the main cytoplasm and is rich in meisosomes (yellow). b) A transverse section of an adult without the internal organ highlight the body wall muscles in red, with the two different regions of the epidermis (sand) and the seam cell (white). Note that the cortical cytoskeleton (brown) is disorganized in the lateral epidermis (yellow), but organized below the circumferential furrows in the dorso and ventral lateral epidermis (blue). c) Hemidesmosomes attach the muscles to the cuticle through basal and apical components linked by intermediate filaments (IFs). d) Meisosomes are specific to the lateral epidermis and ventral and dorsal ridges; they are parallel stacks of folded plasma membrane (pm); mvb, multivesicular bodies. e) TEM image of a longitudinal section showing the different layers of the cuticle and meisosomes (m) at the junction with the epidermal cell (epi); cuticle (cut), furrow (f), mitochondria (mit). Scale bar 200 nm. From Aggad *et al*. (2023).

Prior to its release during molting, the cuticle must be tightly connected to the apical surface of the epidermis. One way that this could occur is through matrix components that contain transmembrane domains, which is potentially the case for many cuticle collagens (*Cuticle collagen maturation*). Circumferential furrows might also serve as sites of transient matrix-cytoskeletal attachment during cuticle formation (Priess and Hirsh 1986; Costa *et al.* 1997; Aggad *et al.* 2023) (see above). In addition, two distinct epidermal structures, HDs and meisosomes, have been implicated in maintaining cuticle connections in different body regions (Fig. 12).

#### Hemidesmosomes

HDs are present in dorsolateral and ventrolateral regions of the epidermis that overlie body muscle (Francis and Waterston 1991; Pasti and Labouesse 2014; Fu *et al.* 2017) (Fig. 12a, c). They consist of both apical and basal pools of the spectraplakin VAB-10 linked by intermediate filaments (IFs) that span the entire epidermis (which is very thin in these regions—see Fig. 12b) (Karabinos *et al.* 2001; Bosher *et al.* 2003). Transmembrane proteins LET-805/myotactin (located basally) and MUP-4 and MUA-3 (located apically) are thought to connect VAB-10 to the underlying basement membrane and muscle or to the overlying cuticle, respectively, though direct biochemical interactions have not yet been demonstrated (Hresko *et al.* 1999; Bercher *et al.* 2001; Hong *et al.* 2001; Pasti and Labouesse 2014). Loss of either MUP-4 or MUA-3 causes the cuticle to separate from the epidermis specifically over muscle regions. HDs are analogous to vertebrate HDs, which also contain spectraplakin and IFs and link matrix to cells, but they are otherwise different in composition (Osmani and Labouesse 2015). MUP-4 and MUA-3 are related to vertebrate matrilins, which can bind collagens and may link them to other matrix factors (Paulsson and Wagener 2018; Li *et al.* 2020).

By linking body muscle to the cuticle exoskeleton, HDs may function like tendons to couple muscle contractions for locomotion (Francis and Waterston 1991; Hahn and Labouesse 2001). HDs also relay muscle-derived forces to help drive embryonic elongation beyond the 2-fold stage (Zhang *et al.* 2011). Because VAB-10/spectraplakin has domains for binding IFs, actin, and microtubules, it could serve as a mechanical hub to transmit tension among these different cytoskeletal networks and various transmembrane proteins, including putative aECM-interacting factors like MUP-4 and MUA-3 (Suman *et al.* 2019). However, the specific connections between HDs and either precuticle or cuticle aECM components remain to be determined.

Another unresolved question concerns the precise relationship of HDs to cuticle furrows and the dynamic circumferential AFBs described above. Spectrin and actin were suggested to pattern the first HDs during embryogenesis (Wang *et al.* 2020). Disruption of HDs by knockdown of *mup-4* or *ptc-3* can induce innate immune signaling through STA-2/STAT; however, these worms are very sick and stage specific targeted abbrogation will be needed to understand the role of HDs in triggering the innate response (Dierking *et al.* 2011; Zhang *et al.* 2015; Zugasti *et al.* 2016; Wang *et al.* 2023).

#### Meisosomes

Meisosomes are folded apical membrane structures that are mainly present in dorsal, ventral, and lateral regions of the epidermis that do not overlie body muscle and do not contain HDs (White *et al.* 1986; Liégeois *et al.* 2006; Aggad *et al.* 2023) (Fig. 12, a, b, d, e). They are also found in some interfacial tubes such as the excretory duct (Kolotuev *et al.* 2009). As observed by TEM, each meisosome consists of up to 30 folds, approximately 200–400 nm deep, with very regular 20 nm spacing between them. Cuticle extends into the outer-facing folds, which provide a large surface area that may serve as attachment points. In furrowless mutants, meisosomes are smaller and the cuticle frequently detaches from the epidermis, specifically outside of the muscle regions (Aggad *et al.* 2023).

Meisosomes (multifold eisosomes) are so-named based on morphological similarity to yeast eisosomes, which are single membrane folds that sense and respond to changes in the yeast cell wall (Moseley 2018). In addition to mediating cuticle attachment, meisosomes define membrane subdomains rich in PH-domain binding sites and could also be sites of signaling to sense and relay information about cuticle damage (Aggad *et al.* 2023). Another possibility is that they play roles in secretion or assembly of aECM components, since they are marked by VHA-5, a V0 subunit of the vacuolar ATPase involved in apical secretion and lumen acidification (Liégeois *et al.* 2006; Kolotuev *et al.* 2009; Aggad *et al.* 2023).

### Molting

Molting is the process by which the old cuticle is removed at the end of each larval stage. Molting has been reviewed comprehensively (Lažetić and Fay 2017a), so here we highlight only some key points and recent advances. There are three major stages of molting as classically defined (Singh and Sulston 1978; Frand *et al.* 2005). We now know that the process of building the new precuticle and cuticle begins well before these recognized stages (Figs. 1 and 2), so in reality larvae are almost always either preparing to molt or molting.

#### Lethargus

When worms are viewed through a dissecting microscope, the first sign of molt initiation is a decrease in movement and feeding. This period of behavioral quiescence, termed lethargus, is a developmentally timed sleep-like state proposed to conserve energy to dedicate to the large metabolic requirements of new cuticle production (Raizen *et al.* 2008).

#### Apolysis

Apolysis is the visible separation of the old and new cuticles, which begins at the mouth and tail before spreading to the rest of the body. Apolysis begins only once the new cuticle is fairly well developed underneath the old one (Fig. 2c). By this time, connections of the plasma membrane to the old cuticle must have been lost and connections to the new matrix established. Apolysis may involve destruction of remaining links between the old and new cuticles or of precuticle matrix in this intervening region. During apolysis, some secreted components of the precuticle accumulate in the space between cuticles (Forman-Rubinsky *et al.* 2017).

#### Ecdysis

Ecdysis is the final process of actual cuticle shedding and is accompanied by the resumption of movement and twisting behaviors that assist in shedding. Many mutants defective in molting (Mlt) have defects at this stage and are easily recognized by the bits of cuticle that remain hanging onto or wrapped around parts of the body (Yochem *et al.* 1999, 2015; Frand *et al.* 2005).

#### Control of molting

Many gene products are required for molting, but we still have a limited understanding of its cell biology (Frand *et al.* 2005; Lažetić and Fay 2017a). No molting hormone equivalent to insect ecdysone has been identified in *C. elegans*, despite the requirements for multiple nuclear hormone receptors such as NHR-23 and NHR-25 (Gissendanner *et al.* 2004; Hayes *et al.* 2006; de Oliveira *et al.* 2019; Patel *et al.* 2022; Johnson *et al.* 2023; Kinney *et al.* 2023). The heterochonic miRNA pathway and transcription factors GRH-1/Grainyhead, BLMP-1/BLMP, and MYRF-1/MYRF may act with these NHRs and LIN-42/Period within the molting clock (Stec *et al.* 2021; Tsiairis and Großhans 2021; Hauser *et al.* 2022; Patel *et al.* 2022; Kinney *et al.* 2023; Meeuse *et al.* 2023; Xu *et al.* 2024). The LDL receptor-like protein LRP-1/megalin is one key molting regulator proposed to affect cholesterol transport and steroid hormone synthesis (Yochem *et al.* 1999). Some trafficking proteins important for molting, such as conserved NEKL kinases and ANK repeat proteins, appear to promote clathrin-dependent endocytosis of LRP-1, but might also affect trafficking of other molting regulators or matrix factors (Kang *et al.* 2013; Lažetić and Fay 2017b; Joseph *et al.* 2020, 2022, 2023).

Most proteins directly involved in molting should be in the matrix. One important recent insight is the requirement for precuticle, which forms between the old and new cuticles and is required for complete ecdysis (*The precuticle*). Several other secreted proteins and transmembrane proteins, including many patched-related proteins, are among the large set of factors identified in RNAi or mutant screens for molting defective (Mlt) phenotypes (Frand *et al.* 2005; Zugasti *et al.* 2005; Hao, Mukherjee *et al.* 2006; Fritz and Behm 2009; Meli *et al.* 2010; Gissendanner and Kelley 2013). Several proteases (NAS-36, NAS-37) and putative protease inhibitors (BLI-5, MLT-11) are required for molting, though their specific substrates are unknown (Davis *et al.* 2004; Suzuki *et al.* 2004; Frand *et al.* 2005; Page *et al.* 2006; Stepek *et al.* 2010, 2011; Ragle *et al.* 2022). Interestingly, one molting regulator is ACN-1, a homolog of the angiotensin converting enzyme ACE2, a carboxypeptidase that also serves as the key receptor for SARS-CoV-2 viral entry (Brooks *et al.* 2003; Pringle and Philp 2023). ACN-1 was suggested to lack catalytic activity based on sequence features (Brooks *et al.* 2003), but this is inconsistent with more recent reports that an ACE inhibitor can mimic some phenotypes of *acn-1* RNAi (Kumar *et al.* 2016; Egan *et al.* 2024). Finally, removal of disulfide bonds from collagens or other matrix factors is important for both apolysis and ecdysis (Stenvall *et al.* 2011) and is discussed below. It will be important to identify the substrates of the various enzymatic factors and to test if changes in their expression or activity actually drive the molting process.

### Cuticles of other nematodes

All nematodes have a collagenous body cuticle, but the number and thickness of cuticle layers and the appearance of cuticle substructures vary widely among different clades and species, presumably reflecting adaptations to their different ecological niches (Chitwood and Chitwood 1974; Malakhov 1994; Kiontke and Fitch 2013). Most nematodes have circumferential furrows and annuli, but many, including *Pristionchus pacificus,* also have longitudinal ridges or punctations (circular cuticle bumps) in complex patterns over the epidermis (Hassani-Kakhki *et al.* 2013; Schroeder 2021). Lateral alae ridges, when present, vary in appearance and can run along the entire length of the body or can be only at the head or tail region; there is even one nematode (*Bunonema*) that has alae on only the left side of its body and not the right (Abolafia 2006). Cuticular feeding structures at the buccal region can include tooth-like protrusions, as in *P. pacificus* and many parasitic species, or very elaborately shaped extensions termed probolae, as in *Cephalobina* (Nadler *et al.* 2006; Susoy *et al.* 2015; Sun *et al.* 2023). Male mating structures also vary considerably (Sudhaus and Fitch 2001; Stevens *et al.* 2019). While classically several such differences were used as criteria for phylogenetic groupings, molecular information has revealed that cuticle features can evolve rapidly and has identified many examples of convergent evolution (Nadler *et al.* 2006; Kiontke *et al.* 2023). With our growing understanding of cuticle content and substructure development in *C. elegans*, the time is ripe for comparative genetic studies of cuticle trait evolution.

The cuticles of parasitic nematodes have some particularly interesting specializations to benefit their unique lifestyles. Infective L3 larvae generally resemble *C. elegans* dauer larvae (Crook 2014; Lok *et al.* 2022), but often this L3 remains ensheathed in the old L2 cuticle until it enters the host (Ondrovics *et al.* 2016). Exsheathment, the process of shedding the L2 cuticle occurs in response to increased temperature and other host signals (Rogers and Sommerville 1957). Hookworms have multiple sharp teeth or “cutting plates” that allow them to embed within the intestinal lining of their hosts (Kiontke and Fitch 2013). The hookworm *Nippostrongylus brasiliensis* also stores hemoglobin within the fluid medial layer of its adult cuticle, giving the worms a bright red appearance; this hemoglobin is thought to increase oxygen availability to help the parasite survive within the host intestine (Sharpe and Lee 1981; Blaxter *et al.* 1994). A better understanding of parasite cuticle and molting could lead to practical applications for controlling many medically and agriculturally damaging nematodes.

## Cuticle collagen maturation and collagen-modifying enzymes

**Fig. 13.**
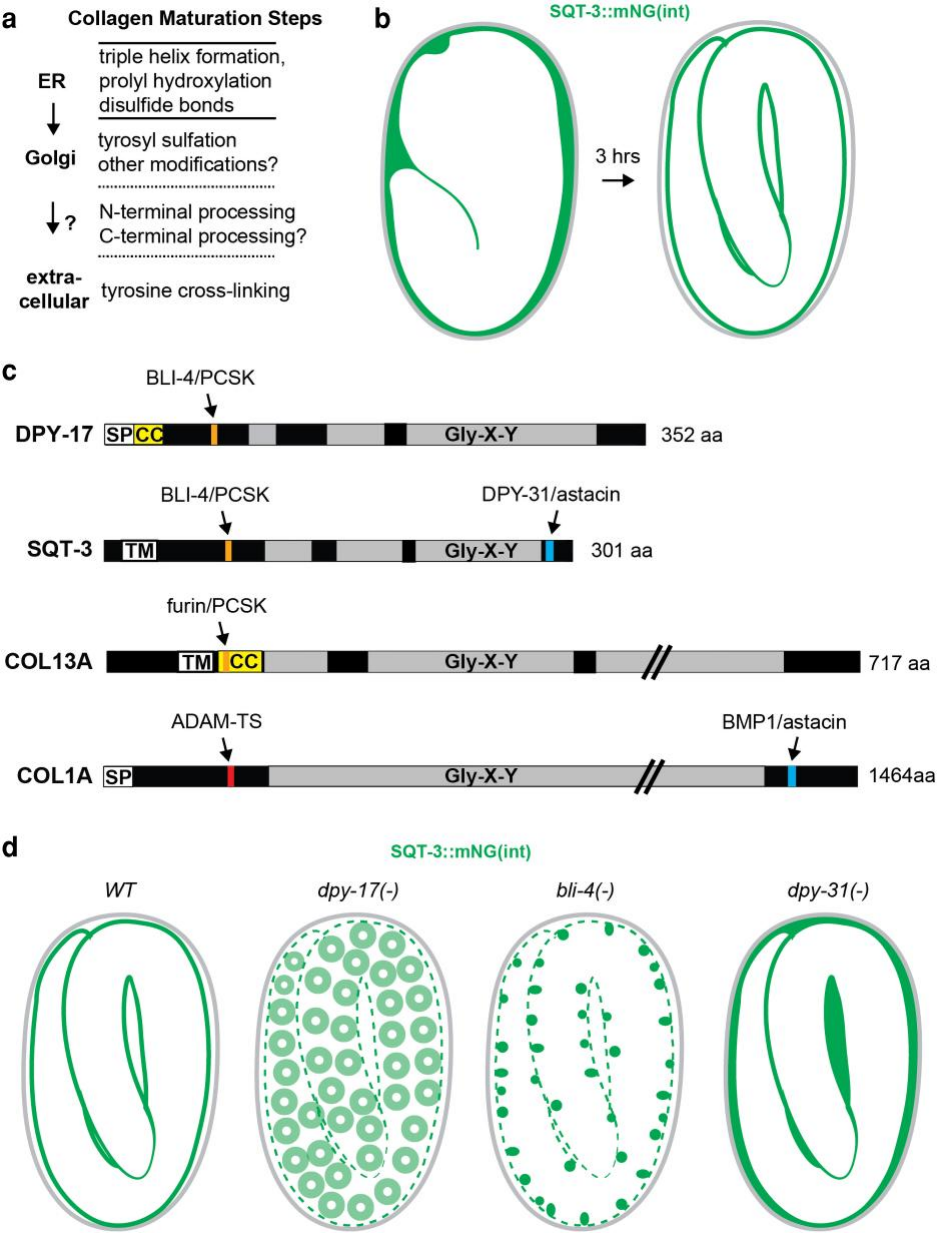
Collagen maturation steps in *C. elegans* and vertebrate collagens. a) Major steps of cuticle collagen maturation, with cellular sites indicated. See text and Supplementary Table 3 for specific genes/enzymes involved. There is still much debate in the literature about where in (or out of) the cell N- and C-terminal cleavage of COL1 occurs (Hellicar *et al.* 2022), nor do we know this information for cuticle collagens. b) Cuticle collagen maturation is temporally controlled. SQT-3::mNG(int) is secreted into the extraembryonic space before the 1.5-fold stage, but it only detectably assembles into cuticle ∼3 h later (Birnbaum *et al.* 2023). The mNeonGreen tag is located internally, soon after the BLI-4/PCSK-dependent cleavage site. c) Structural comparison of *C. elegans* and vertebrate collagens and their proteolytic cleavage sites. Ce DPY-17 is an example of a conventionally secreted cuticle collagen with a signal peptide (SP), while Ce SQT-3 is a predicted type II transmembrane (TM) protein. Human COL-13A is a transmembrane MACIT collagen (Tu *et al.* 2015) and COL1A is the classic type I fibrillar collagen (Revell *et al.* 2021). Gray boxes indicate the Gly-X-Y motifs, which are interrupted in cuticle collagens and MACITs. Black boxes indicate noncollagenous domains. Both DPY-17 and COL13A have predicted coiled-coil (CC, yellow) domains N-terminal to their collagen domains (Ludwiczak *et al.* 2019), and both and SQT-3 have sites for furin/PCSK-dependent cleavage (orange), whereas COL1A is cleaved by ADAM-TS. Only SQT-3 and COL1A have evidence for astacin protease cleavage (blue) at their C-termini. Note that proteins and domains are not drawn to scale. Genbank accession #s: DPY-17: NP_498086.1; SQT-3: NP_001256412.1; COL13A; NP_001123575; COL1A1, P02452. d) In the embryo, loss of DPY-17 collagen, BLI-4/PCSK, or DPY-31/astacin disrupts SQT-3 maturation prior to ER export, apical secretion, or cuticle assembly, respectively (Birnbaum *et al.* 2023). Note that these are temporary or partial blocks, because by hatch some SQT-3 does incorporate into the cuticle of these mutants.

Collagen maturation is a multi-step process involving association of individual peptides to form a triple helix, prolyl hydroxylation to stabilize the helix, formation of disulfide bonds, various other post-translational modifications, removal of noncollagenous N- and C-terminal domains, and eventually covalent cross-linking to form higher order fibrils or networks in the extracellular environment (Fidler *et al.* 2018; Karamanos *et al.* 2021; Revell *et al.* 2021) (Fig. 13a). Many of these steps have been worked out through studies of vertebrate type I fibrillar collagen but are less well characterized for other collagen types. Furthermore, even for type I collagen, many questions remain about how these steps are controlled in vivo to ensure that matrix assembly occurs only at the right time and place.

*Caenorhabditis elegans* cuticle collagens are distinct from any of the 28 collagen types defined in vertebrates (Karamanos *et al.* 2021), but as described below and shown in Fig. 13c, they are in several ways more similar to the lesser known transmembrane collagens than they are to the more extensively studied type I fibrillar collagen or type IV basement membrane collagen. Their small size is also reminiscent of collectins and other “collagen-related” proteins found in mammalian aECMs (Sorensen 2018; Murugaiah *et al.* 2020), though they lack the other domains found in those proteins. *Caenorhabditis elegans* offers several advantages for studying collagens, including the ability to visualize collagens in vivo during their maturation, trafficking, and assembly. Many gene products that impact cuticle collagen assembly have been identified by homology to vertebrate enzymes or based on their embryonic lethal, Dpy, Bli, or Mlt mutant phenotypes (Supplementary Table 3) (Page *et al.* 2014). Below we highlight similarities and differences in the known ways that vertebrate collagens and *C. elegans* cuticle collagens are regulated.

### Cuticle collagens include a mix of predicted transmembrane and secreted proteins

Approximately two-third of cuticle collagens are predicted to be produced as type II transmembrane (TM) proteins with a short cytosolic N-terminus followed by a TM domain and an extracellular collagenous C-terminus (Fig. 13c) (Teuscher *et al.* 2019; Birnbaum *et al.* 2023). Both the TM and conventionally secreted cuticle collagens have potential furin cleavage sites (RxxR) near their N-termini; this could allow release of the collagenous domains from the transmembrane forms at some point along the secretory pathway or at the cell surface. All cuticle collagens have interrupted triple helices, as described further below. The type II transmembrane topology, furin site, and interrupted triple helix features are all similar to those of the human MACITs (membrane associated collagens with interrupted triple helices), which include Collagen XIII, XXIII, and XXV (Tu *et al.* 2015; Wakabayashi 2020; Karamanos *et al.* 2021) (Fig. 13c). Cuticle collagens lack a conserved C-terminal domain found in true MACIT orthologs such as the neuromuscular junction collagen COL-99. Nevertheless, the other shared features suggest there could be parallels in the cell biology and regulation of cuticle collagens and MACITs.

The MACIT COL13 is mutated in human patients with congenital myasthenic syndrome type 19 (CMS19), a disease of the neuromuscular junction and skeleton (Logan *et al.* 2015). Studies of this collagen suggest that both the TM form and the cleaved secreted form have important functions (Härönen *et al.* 2017; Kemppainen *et al.* 2022); the same could be true of *C. elegans* TM cuticle collagens.

### Cuticle collagen secretion can precede cuticle assembly by several hours

New tools for collagen visualization are providing important insights into their secretion and matrix assembly patterns. SQT-3, a predicted TM collagen, does appear to be released into the extracellular environment in a cleavage-dependent manner (Birnbaum *et al.* 2023) (see below). In the embryo, where the eggshell constrains the diffusion of apically secreted proteins, SQT-3, DPY-17, and other cuticle collagens accumulate in the extraembryonic space prior to the 1.5-fold stage, several hours before cuticle formation (Fig. 13b) (Birnbaum *et al.* 2023). In adults, the coalescence of BLI collagens into struts also occurs over a period of an hour or more (Adams *et al.* 2023). These observations indicate that assembly is not instantaneous upon secretion and that there must be other temporally controlled regulatory steps.

### The Gly-X-Y motif and collagen triple helix formation

The hallmark of all collagens is a repeating Gly-X-Y sequence, where X and Y are often proline or hydroxyproline. These Gly-X-Y domains allow collagens to assemble into the triple helices that give them their exceptional mechanical strength (Fidler *et al.* 2018). Triple helices can be either homotrimers or heterotrimers of different related collagen chains. Type I collagen has a long uninterrupted stretch of >350 Gly-X-Y repeats, which allows it to form long rod-like fibrils (Revell *et al.* 2021). Collagen IV and other nonfibrillar collagens have imperfect or interrupted stretches of Gly-X-Y repeats, which is generally thought to lead to a more bent or flexible network structure (Fidler *et al.* 2018). *Caenorhabditis elegans* cuticle collagens typically have two or three blocks of Gly-X-Y repeats separated by noncollagenous spacers (Teuscher *et al.* 2019). Some of these Gly-X-Y blocks are quite short (∼10 repeats) while the main block in each collagen is somewhat longer (37–43 repeats) but has additional small interruptions. These features are most consistent with a mesh-like cuticle collagen network.

Triple helix formation occurs in the endoplasmic reticulum (ER) and is a very early and essential step of collagen matrix assembly (Fig. 13a) (Fidler *et al.* 2018). In type I collagen, triple helix formation initiates through interactions at the noncollagenous C-terminus and then proceeds in zipper-like fashion toward the N-terminus, whereas in transmembrane collagens the process occurs in the opposite direction (Snellman *et al.* 2000; Areida *et al.* 2001). For *C. elegans* cuticle collagens, we do not yet know the direction of triple helix assembly, but the frequent presence of a TM domain or coiled-coil domain near their N-termini is suggestive of an N to C assembly direction, since both types of domains have been shown to act as nucleation centers in other collagens (Bulleid *et al.* 1997; Latvanlehto *et al.* 2003; McAlinden *et al.* 2003).

We do not know which cuticle collagens form homo- vs hetero-trimers. Several functionally-related cuticle collagens rely on each other for their trafficking or stable accumulation/localization (e.g. SQT-3 and DPY-17, Fig. 13d) (McMahon *et al.* 2003; Novelli *et al.* 2006; Chandler and Choe 2022; Birnbaum *et al.* 2023), but their differing sequences and numbers of GLY-X-Y repeats make it unlikely that these form heterotrimers. Instead, it has been proposed that these partner collagens form other higher order multimers while still intracellular (Novelli *et al.* 2006).

### Prolyl 4-hydroxylation stabilizes collagen triple helices

Proline hydroxylation within collagen Gly-X-Y repeats occurs in the ER and is important to stabilize the triple helix structure (Salo and Myllyharju 2021). The prolyl 4-hydroxylase (P4H) enzyme required for this modification is a tetramer with two alpha and two beta subunits. In humans, insufficient P4H activity caused by vitamin C deficiency leads to scurvy (Barnes 1975). In *C. elegans*, there are two different P4H alpha subunits (DPY-18 and PHY-2) and three potential beta subunits (PDI-1,2,3), of which PDI-2 is essential (Myllyharju *et al.* 2002). Loss of *pdi-2* (via RNAi to target both maternal and zygotic gene expression) or of both *dpy-18* and *phy-2* together leads to an embryonic lethal phenotype where embryos retract and explode after initiating elongation (Friedman *et al.* 2000; Winter and Page 2000; Winter *et al.* 2007). Single mutants for *dpy-18* or *phy-2* are viable but have reduced levels of hydroxyproline (Friedman *et al.* 2000; Winter and Page 2000).

### Disulfide bonds link collagen chains and triple helices

Disulfide bonds involving cysteines adjacent to the Gly-X-Y region also form in the ER and are thought to aid in triple helix formation or stability and also in cross-linking between triple helices to form higher order structures (Barth, Kyrieleis *et al.* 2003; Barth, Musiol *et al.* 2003). Protein disulfide isomerases (PDIs), which function as part of the P4H enzyme, also can catalyze formation or rearrangements of these disulfide bonds (Bechtel and Weerapana 2017).

Most *C. elegans* cuticle collagens have three clusters of cysteines surrounding the two major Gly-X-Y regions (Johnstone 2000; Teuscher *et al.* 2019). Mutation of one such cysteine in SQT-1 causes a neomorphic Roller phenotype (whereas null mutants appear normal) and prevents tyrosine cross-linking of SQT-1 triple helices (Yang and Kramer 1999) (see below), suggesting that disulfide bonding may facilitate tyrosine cross-linking. Removal of disulfide bonds occurs during molting (Stenvall *et al.* 2011) (see below).

### Collagen trafficking from the ER to Golgi

Like most secreted and transmembrane proteins, collagens are transported from the ER to the Golgi in compartments marked by coat complex II (COPII) (Aridor 2022). Cuticle collagen secretion therefore requires COPII components such as Sec23 as well as transport protein particle (TRAPP) complex components that tether COPII vesicles to their destination compartments (Roberts *et al.* 2003; Thein *et al.* 2003; Zhang *et al.* 2020). The large size of vertebrate type I collagen triple helices (up to 400 nm long) makes them too large to fit in conventional COPII-coated vesicles (which are <100 nm in diameter); instead their transport requires especially large COPII-marked tubular compartments whose formation involves specialized ER exit site factors such as TANGO1 (Aridor 2022). However, *C. elegans* cuticle collagens are generally much smaller than type I collagen, and no TANGO ortholog has been identified in the worm. Instead, RNAi screens for defects in secretion of the adult cuticle collagen COL-19 have identified conserved transmembrane proteins TMEM-39 and TMEM-131 that appear to link COL-19 to COPII or TRAPP components, respectively (Zhang *et al.* 2020, 2021). Consistent with a requirement in ER-to-Golgi transport, loss of *tmem-39* blocked COL-19 secretion at a step upstream of processing and cross-linking. Interestingly, TMEM-39 and TMEM-131 are required for secretion of only a subset of *C. elegans* collagens, suggesting some cargo specificity. Knockdown of TMEM-131 or TMEM-39 orthologs in mammalian cells or in Drosophila also disrupted collagen secretion, suggesting a conserved function (Zhang *et al.* 2020, 2021).

### Tyrosine sulfation of collagen

Once in the Golgi, collagens can undergo additional post-translational modifications such as tyrosine sulfation (Stone, Chuang *et al.* 2009; Igreja and Sommer 2022). *tpst-1* encodes a conserved transmembrane tyrosylprotein sulfotransferase that localizes to the trans-Golgi network. It was identified as a suppressor of *rol-6(gf)* and other Roller mutants, can modify tyrosines on ROL-6 in vitro, and is required for ROL-6 secretion (Kim *et al.* 2005, 2010). RNAi knockdown of *tpst-1* also caused larval lethal, Dpy, and molting defective phenotypes, consistent with broad effects on aECM.

### Collagen trafficking to the apical surface

Upon leaving the Golgi, cuticle collagens must be routed toward the apical plasma membrane and must pass through appropriate other secretory compartments for the further processing steps described below. However, the specific pathways required for these late steps of cuticle collagen secretion have not been identified yet. Interestingly, electron micrographs revealed the presence of numerous extracellular vesicles (EVs) within the developing adult cuticle (Katz *et al.* 2022). Furthermore, proteomic analysis has identified several cuticle collagens as potential EV cargos (Nikonorova *et al.* 2022). These data raise the interesting but still speculative possibility that cuticle collagen trafficking may involve multivesicular bodies and/or EVs. Multivesicular bodies and VHA-5 have been tied to the secretion of other apparent aECM factors, the Hh-r proteins (Liégeois *et al.* 2006).

### N- and C-terminal processing promote collagen secretion or assembly

At some point along the secretory pathway, after triple helix formation but before matrix assembly, some collagens are cleaved to remove their N- and/or C-terminal domains (Revell *et al.* 2021). In human type I collagen, these cleavages are carried out by ADAM-TS and BMP1/astacin proteases, respectively (Fig. 13c), and defects in cleavage cause connective tissue disorders such as Ehler Danlos syndrome or osteogenesis imperfecta (Lichtenstein *et al.* 1973; Lindahl *et al.* 2011; Martínez-Glez *et al.* 2012; Bekhouche and Colige 2015). N-terminal cleavage affects fibril structure (Holmes *et al.* 1993), while C-terminal cleavage is believed to be a key regulatory event that happens in a late secretory compartment or at the cell surface, ensuring that fibrillogenesis occurs only once the collagen reaches the extracellular space (Kadler *et al.* 1987; Revell *et al.* 2021). However, not all collagen types undergo cleavage and, for those that do, the roles of cleavage are not as well defined as for collagen type I. For example, mutational analysis of the MACIT COL13 suggests that N-terminal cleavage is functionally important, but it is not known if cleavage has roles in maturation beyond shedding and there is no evidence for C-terminal cleavage (Tu *et al.* 2015; Härönen *et al.* 2017; Wakabayashi 2020; Kemppainen *et al.* 2022).

#### N-terminal processing

Many *C. elegans* cuticle collagens appear to be N-terminally cleaved by BLI-4, a furin-related member of the proprotein convertase subtilisin/kexin (PCSK) family (Thacker *et al.* 1995) (Fig. 13, c and d). *bli-4* null mutants are embryonic lethal and retract after fully elongating, similar to mutants for the essential cuticle collagen SQT-3 (Priess and Hirsh 1986; Birnbaum *et al.* 2023). Most cuticle collagens contain a consensus furin cleavage site (CFCS), usually RxxR, located either near the N-terminus (for secreted collagens) or shortly after the transmembrane domain (for TM collagens) (Yang and Kramer 1994; Teuscher *et al.* 2019). Mutations in this consensus sequence have been reported for the collagens BLI-1, BLI-6, DPY-5, DPY-10, DPY-17, ROL-6, SQT-1, SQT-2, and SQT-3—and in all cases the mutations cause disruptions to cuticle structure, indicating functional importance (Kramer and Johnson 1993; Levy *et al.* 1993; Yang and Kramer 1994; Thacker *et al.* 2006; Adams *et al.* 2023; Birnbaum *et al.* 2023). In the case of SQT-3 (a TM collagen) and DPY-17 (a secreted collagen), visualization of the mutant collagens with a fluorescent tag revealed that the collagens were inefficiently secreted and instead formed intracellular puncta at or close to the apical membrane (Birnbaum *et al.* 2023). Similar results were seen with the wild type collagens in a *bli-4* mutant background (Birnbaum *et al.* 2023) (Fig. 13d). These observations suggest that cuticle collagen N-terminal cleavage promotes a late step in apical secretion and/or prevents premature aggregation of both TM and secreted collagens prior to their extracellular release.

*bli-5* and *mlt-11* encode secreted Kunitz domain proteins (Frand *et al.* 2005; Page *et al.* 2006; Ragle *et al.* 2022), which are typically thought to act as serine protease inhibitors (Mishra 2020); as such, they could potentially influence BLI-4 activity. However, BLI-5 actually enhanced serine protease activity in vitro, suggesting it could be a protease co-factor instead (Stepek *et al.* 2010). A gain-of-function allele of *mlt-11* causes a Rol phenotype (Rich *et al.* 2022).

#### C-terminal processing

Whether C-terminal cleavage occurs remains uncertain for most cuticle collagens. *Caenorhabditis elegans* does have a large family of astacin proteases related to BMP1 (Park *et al.* 2010), some of which are required for molting (NAS-36, NAS-37) or body shape (DPY-31) (Davis *et al.* 2004; Novelli *et al.* 2004; Suzuki *et al.* 2004; Stepek *et al.* 2010, 2011). Furthermore, SQT-3 contains a C-terminal consensus astacin site that appears to be cleaved by the astacin protease DPY-31 (Fig. 13a), based on the isolation of specific *sqt-3* missense mutations acting as *dpy-31* suppressors (Novelli *et al.* 2004). *dpy-31* mutants also have delayed SQT-3 matrix incorporation (Fig. 13d), although some residual incorporation eventually occurs (Novelli *et al.* 2006; Birnbaum *et al.* 2023). However, most cuticle collagens lack an obvious astacin cleavage site and C-terminally tagged forms of many collagens mark the mature cuticle (Miao *et al.* 2020; Zhang *et al.* 2020; Adams *et al.* 2023; Aggad *et al.* 2023; Birnbaum *et al.* 2023). Therefore, C-terminal cleavage may occur in only a subset of cuticle collagens.

### Covalent cross-linking of collagen triple helices generates higher order matrix structures

Within extracellular matrix, collagen triple helices are covalently linked together to form larger diameter fibrils or networks with appropriate mechanical properties. In vertebrate type I collagen, extensive cross-linking occurs at lysine residues that are located both N- and C-terminal to the Gly-X-Y domain (Yamauchi *et al.* 2019). Once type I collagens reach the extracellular environment, these lysines are deaminated by lysyl oxidase (LOX) to generate reactive aldehydes that eventually form the cross-links. Cross-linking efficiency can be influenced by collagen binding matrix proteins such as members of the small leucine-rich proteoglycan family that share domains with eLRRon proteins (Kalamajski and Oldberg 2010; Mancuso *et al.* 2012; Kalamajski *et al.* 2014).

*Caenorhabditis elegans* cuticle collagens are cross-linked at tyrosine residues rather than at lysines. It is proposed that the peroxidase MLT-7 and the dual oxidase (DUOX) BLI-3 (a multi-pass transmembrane protein) catalyze oxidation of tyrosines using hydrogen peroxide generated by the NADPH oxidase activity of BLI-3 (Thein *et al.* 2009; van der Hoeven *et al.* 2015). Cross-linking further requires the DUOX chaperone DOXA-1 and the tetraspanin TSP-15, which both bind BLI-3 and are putative regulators of BLI-3 activity at the plasma membrane (Moribe *et al.* 2012). Loss of *mlt-7* or partial loss of *bli-3*, *doxa-1,* or *tsp-15* result in larval lethal, molting, blistering, and body shape defects consistent with a weakened cuticle. Loss of other peroxidases like HPX-2 and SKPO-1 also leads to cuticle defects (Tiller and Garsin 2014; Liu *et al.* 2019).

### Molting involves removal of disulfide crosslinks

Unlike tyrosine cross-links, disulfide crosslinks are reversible; their removal occurs during molting and involves the thioredoxin and glutathione antioxidant systems, in a thiol-disulfide exchange reaction (Stenvall *et al.* 2011). Disulfide cross-linking status was assessed with the dye Alexa Fluor 488 C5-maleimide, which reacts with extracellular thiol (-SH) groups but not with disulfides. While there was minimal staining of cuticle in the intermolt periods, staining dramatically increased during cuticle shedding. Joint depletion of TRXR-1/thioredoxin reductase and GSR-1/glutathione reductase disrupted molting at a step before disulfide removal. These reductases may act by affecting the oxidation status of glutathione (GSH), since blocking GSH synthesis blocked apolysis (separation of old and new cuticles), while providing exogenous GSH stimulated apolysis. These observations suggest that temporally modulating GSH levels and/or oxidation status could be one key mechanism for regulating molt timing. *dpy-11* encodes another thioredoxin that affects body shape and not molting (Ko and Chow 2002).

### Which maturation steps determine the proper time and place of cuticle collagen assembly?

All of the above maturation steps are important for proper cuticle formation, but which steps are constitutive and which are key regulatory nodes that determine when and where collagens assemble into cuticle? The fact that collagens are initially expressed and secreted from the embryo without assembling indicates that one or more steps must be temporally controlled (Birnbaum *et al.* 2023). For example, perhaps enzymes important for C-terminal cleavage or cross-linking are not activated until the appropriate time in development or initial trafficking routes do not allow collagens to access them. Alternatively, perhaps N-terminal cleavage of TM collagens prevents premature matrix assembly and then must be halted to allow assembly to proceed at the cell surface once appropriate partners are in place. Answering these questions will be important for understanding how the different layers of the cuticle are formed in sequence.

## Outstanding questions and future prospects

The precuticle and cuticle coexist during part of each molt (Fig. 1) and therefore are not completely different aECMs but rather two endpoints of a continuum. They differ in collagen content but share many molecular similarities, such as the presence of ZP proteins. Precuticle is, however, unique as it is transient and endocytosed at each molt cycle. Here, we discuss some of the possible benefits that such a diverse and dynamic aECM might confer to the organism and highlight key questions to be addressed in future research.

### What is the relationship between precuticle and cuticle?

**Fig. 14.**
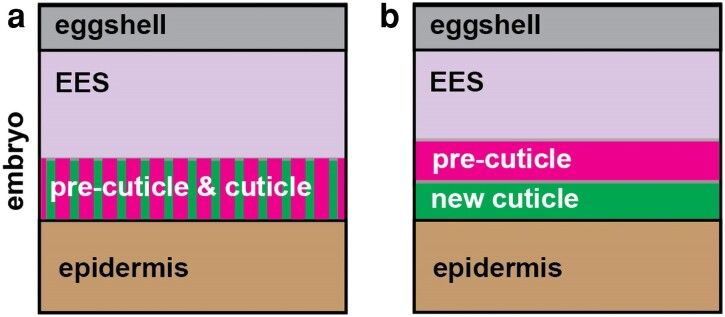
Models for the relationship between the first precuticle and cuticle. a) Is the precuticle a porous structure that lets collagens pass through it and assemble within it? b) Or is the precuticle a separate layer that corrals new collagens below it? (It is also possible that, through phase separation, the aECM transforms from an initial arrangement as in (a) to a later arrangement as in (b). While (a) seems most compatible with subsequent precuticle endocytosis, (b) could explain how the precuticle disrupts interactions between the new cuticle and preexisting matrices (such as the eggshell in embryos or the old cuticle in larvae) to promote successful hatching and molting. EES, extraembryonic space.

Because several precuticle components assemble in the sheath hours before cuticle collagens, it has been proposed that precuticle serves as a temporary scaffold to recruit and pattern those collagens (Forman-Rubinsky *et al.* 2017; Cohen and Sundaram 2020). For example, precuticle might allow newly secreted collagens to infiltrate and assemble within it, or it might corral collagens below it to encourage membrane-proximal assembly (Fig. 14). Newly available collagen reporter fusions will now allow tests of these hypotheses. To date, only mild disruptions to furrow collagen organization have been observed in precuticle mutants (Cohen *et al.* 2021), but the mutant phenotypes suggest that changes to other types of collagens or ZP proteins may be more severe. In particular, the alae and barrier defects of several precuticle mutants (Mancuso *et al.* 2012; Forman-Rubinsky *et al.* 2017; Katz *et al.* 2022) suggest potential effects on cortical and epicuticle layer assembly.

In some cases, the prior cuticle also patterns the new precuticle. For example, several precuticle components specifically mark developing furrows or annuli in wild type larvae, but not in furrowless collagen mutants (Forman-Rubinsky *et al.* 2017; Serra *et al*. 2024; Sonntag *et al.* 2024). This suggests that existing furrows somehow direct appropriate secretion and/or assembly of the next precuticle and cuticle to transmit the furrow pattern to the next stage. Consistent with this idea, imaging of BLI and ROL-6 collagens suggested that furrows are sites of initial aECM secretion (Adams *et al.* 2023; Johnson *et al.* 2023).

Finally, because cuticle assembly is followed shortly by precuticle endocytosis (Birnbaum *et al.* 2023), and precuticle assembly is followed shortly by cuticle apolysis (Forman-Rubinsky *et al.* 2017; Katz *et al.* 2022), some components of each matrix may play an active role in ejecting the prior matrix to drive the molt cycle forward. Such a role could explain the molting defects of some precuticle mutants (Frand *et al.* 2005; Forman-Rubinsky *et al.* 2017).

### How are complex three-dimensional aECM structures constructed outside cells?

Within cells, elaborate cytoskeletal networks and vesicular trafficking routes control the locations of proteins and other materials. But how can cells control the sculpting of matrices in their extracellular environment? For example, how do epidermal collagens form specific substructures such as furrows vs annuli, alae vs valleys, or struts vs uniform layers? These propensities may be determined by intrinsic biophysical properties of the relevant collagens and their abilities to access specific trafficking compartments, interact with other specific matrix factors, or bind localized transmembrane or cytoskeletal partners. For example, strut formation requires the unique N-terminus of BLI-1 and live imaging suggested struts form gradually by liquid–liquid phase separation once BLI-1 and BLI-2 are secreted into the cuticle (Adams *et al.* 2023). Phase separation was shown for the ECM tropoelastin and some pharynx cuticle components (Muiznieks *et al.* 2018; Kamal *et al.* 2022), suggesting it may be widely relevant for aECM layer and substructure formation. Structure/function and imaging studies to test this idea will be facilitated by all the newly developed tools for visualizing collagens and other aECM components.

### What signals or social cues are embedded in aECM?

Another fascinating aspect of *C. elegans* matrix biology is the presence of many divergent Hh-r proteins that appear to serve both structural and signaling roles (*Introduction*). The precuticle Hh-r proteins are ideally poised to serve as temporal signals to influence developmental progression (Kume *et al.* 2019; Chiyoda *et al.* 2021; Emans *et al.* 2023), since they will be released and/or endocytosed at specific timepoints during the molt cycle. On the other hand, cuticle Hh-r proteins may be appropriately positioned to serve as social cues for signaling between animals. In this regard, the presence of GRL-18 at the hermaphrodite vulva opening is particularly intriguing (Serra *et al*. 2024). Furthermore, along with secreted antimicrobial peptides (Martineau *et al.* 2021), several Hh proteins are upregulated after infection and/or important for survival on different pathogens (Mallo *et al.* 2002; Zugasti *et al.* 2014; Liu *et al.* 2021; Zárate-Potes *et al.* 2022). The cysteine cradle protein SPIA-1 is found at furrows and involved in signaling after cuticle damage (Sonntag *et al.* 2024). Surely additional secreted proteins associated with the aECM remain to be discovered.

### How do mechanical properties influence the function of the aECM?

Unlike the rigid cuticle of insects, nematode cuticle is capable of stretching to accommodate growth during larval stages and adulthood. However, there are limitations to this stretching, which has been proposed to be one of the triggers to induce molting (Nyaanga *et al.* 2022). We hypothesize that the precuticle is even more flexible and therefore better able to accommodate large changes in tissue growth and cell shape during embryo morphogenesis. Its periodic presence in the larval intermolt periods may also alter the stiffness of the overlying cuticle and contribute to its stretchable properties. The precuticle also may help maintain body shape during remodeling of epidermal connections from the old cuticle to the new one.

Cuticle stretching allows adult worms to handle water influx during osmotic challenges or desiccation (Urso and Lamitina 2021; Rahimi *et al.* 2022; Sonntag *et al.* 2024). Immune reactions to pathogens like oomycetes have been described to change body stiffness (Osman *et al.* 2018). Functioning as a vibration sensor, cuticle stretching allows mechanosensory neurons to perceive various mechanical loads (Sanzeni *et al.* 2019), and detect sound vibrations (Iliff *et al.* 2021). Different regular structures in the cuticle might account for these different properties. The furrows that exhibit a periodic circumferential pattern in the cuticle, have been shown to impact body stiffness, and could act as a reinforcing material (Aggad *et al*. 2023). The struts are elegant regular orthogonal pillars linking the basal and cortical cuticle but leaving a fluid-filled cushion between them (Adams *et al.* 2023); these likely alter the aECM's mechanical characteristics and have been identified to facilitate sound vibrations (Iliff *et al.* 2021). A combination of super resolution microscopy and biophysics will unravel the mechanobiology of the aECM. The exploration of how spatial arrangements in physical characteristics initiate morphogenesis is an expanding and intriguing area of research across various species (Banavar and Nelson 2023; Yang, Palmquist *et al.* 2023).

### What mechanically connects the aECM to underlying tissues?

Understanding aECM mechanical properties will require better knowledge of how each aECM is mechanically coupled to underlying tissues. In the L4 seam, mechanical connections between cortical actin AFBs and the overlying precuticle matrix may be important to generate proper patterns of matrix delamination and deposition to build the alae (Katz *et al.* 2022) (Fig. 8). HDs play important roles in connecting muscle forces to epidermal morphogenesis during both precuticle and cuticle phases of embryogenesis (Vuong-Brender *et al.* 2017; Lardennois *et al.* 2019). The main lateral epidermis lacks classical HDs, yet is still capable of reacting to injury and inducing an immune response, possibly via disruptions to other mechanosensitive structures such as furrows, struts, or meisosomes (Pujol, Cypowyj *et al.* 2008; Taffoni *et al.* 2020; Adams *et al.* 2023; Aggad *et al.* 2023). Moreover the underlying epidermal cytoskeleton can also react to damage and possibly to changes in tension (Xu and Chisholm 2011; Taffoni *et al.* 2020; Aggad *et al.* 2023). The precise mechanisms linking cuticle damage, tension changes and immune signaling in *C. elegans* remains an active area of research, and may provide answers to more general questions regarding the composition and functions of poorly understood aECMs.

## Supplementary Material

iyae072_Supplementary_Data

## Supplemental Material

Supplemental material available at GENETICS online.
