# Supplementary material for: The Caenorhabditis elegans cuticle and precuticle: a model for studying dynamic apical extracellular matrices in vivo
Source: Genetics. 2024 Jul 12;227(4):iyae072. doi: 10.1093/genetics/iyae072 (PMC11304992; doi:10.1093/genetics/iyae072)
Supplement: iyae072_Supplementary_Data [file iyae072_supplementary_data.zip › Supplemental_Material_Legends_GENETICS-2023-306239.docx]

**Supplementary materials (in Excel file)**

Table S1: Pre-cuticle and cuticle matrix proteins

Table S2: *bah, bus* and *srf* genes required for proper formation or function of the surface coat

Table S3: Gene products involved in collagen maturation and trafficking
